# From Wild to Farm: Gut Bacteriome Differences and Probiotic Potential of *Pantoea Agglomerans* in Two-Spotted Cricket (*Gryllus Bimaculatus*) Rearing

**DOI:** 10.1007/s12602-026-11001-1

**Published:** 2026-04-22

**Authors:** Diego Cruz, Zaki Saati-Santamaria, Luisa Achury-Arrubla, Paula Garcia-Fraile

**Affiliations:** 1 Departamento de Microbiología y Genética, Universidad de Salamanca, Salamanca, Spain; 2 Instituto de Investigación en Agrobiotecnología (CIALE), Universidad de Salamanca, Salamanca, Spain; 3 Unidad de Excelencia Producción, Agrícola y Medioambiente (AGRIENVIRONMENT), Universidad de Salamanca, Salamanca, 37185 Spain; 4 Laboratory of Fungal Genetics and Metabolism, Institute of Microbiology, The Czech Academy of Sciences, Prague, Czech Republic; 5 Associated Research Unit of Plant-Microorganism Interaction, Universidad de Salamanca-IRNASA-CSIC, Salamanca, Spain; 6 Plaza Doctores de La Reina S/N. Edificio Departamental, Laboratorio 237, Salamanca, Spain

**Keywords:** Edible insects, *Gryllus bimaculatus*, Gut microbiota, Probiotics, Genomics, *Pantoea agglomerans*

## Abstract

The gut microbiome plays a crucial role in insect nutrition and performance, yet its targeted exploitation in cricket farming remains underexplored. Here, we combined gut microbiota profiling of wild and farmed *Gryllus bimaculatus* with probiotic testing of host-derived bacterial isolates to explore microbiome-informed strategies for sustainable cricket farming. Wild crickets exhibited higher Shannon diversity but lower phylogenetic diversity than farmed counterparts. Wild populations were enriched in *Oscillospiraceae* and *Christensenellaceae* families, while farmed crickets showed higher abundance of *Parabacteroides*. From 199 bacterial isolates, wild populations showed higher frequencies of uricolytic capabilities (44% vs. 31%), related to nitrogen recycling, while farmed crickets had more pectinolytic isolates (70% vs. 50%), linked to plant fiber degradation. *Pantoea agglomerans* I53BLB, which demonstrated broad enzymatic capabilities, was selected for probiotic evaluation; we further provide its genome sequence and analysis to contextualize its metabolic and probiotic potential. A feeding experiment with a 2 × 3 factorial design (two diets × three probiotic treatments, *n* = 10 replicates per group) compared control chicken feed versus a high-fiber diet formulated with agricultural by-products, each supplemented with water, live or heat-inactivated *P. agglomerans*. A significant diet × probiotic interaction was observed for weight gain (χ² = 18.8, *p* = 0.0021) and adult emergence (χ² = 17.7, *p* = 0.0033). Live *P. agglomerans* enhanced performance only when combined with the high-fiber diet, with individuals reaching a mean wet weight of 0.602 g compared to 0.451 g (heat-inactivated, *p* = 0.035) and 0.427 g (water control, *p* = 0.003), and a significantly higher adult emergence rate (37%) compared to all other treatment combinations (13%, *p* < 0.05), suggesting a symbiotic effect likely related with carbohydrate digestion. No effects were observed on survival or reproductive output. Notably, the high-fiber diet alone performed comparably to commercial feed, suggesting potential for sustainable cricket production using agricultural by-products. These findings demonstrate the feasibility of microbiome informed probiotic strategies to enhance cricket farming efficiency while reducing feed costs.

**Graphical Abstract:**

**Figure:**
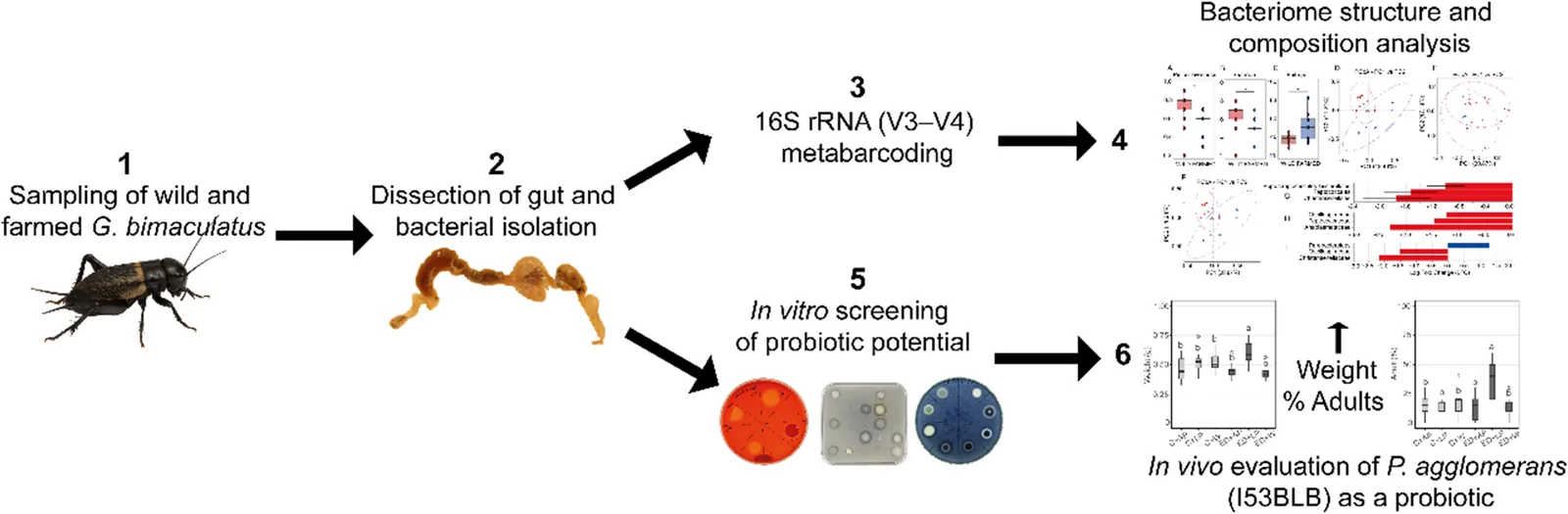

**Supplementary Information:**

The online version contains supplementary material available at 10.1007/s12602-026-11001-1.

## Introduction

*Gryllus bimaculatus* is widely distributed across the tropical and subtropical regions in Europe, Africa, and Asia [1] and is considered one of the most significant edible insects in Asia due to its high nutritional value, short life cycle, and superior size and economic yield compared to other cricket species [2]. Although not yet approved by the European Food Safety Authority (EFSA) as a novel food in Europe, its potential remains substantial, particularly due to its natural distribution in the Mediterranean region, which offers a continuous source of wild individuals for the genetic enrichment of captive populations. In addition, it has a long-standing history of safe consumption in Asia, a high nutritional value, efficient feed conversion, and a low environmental impact, making it highly aligned with current sustainability and food security goals in the EU.

Nutritionally, *G. bimaculatus* contains approximately 54–58% crude protein, 6.90% crude fiber, 26.90% fat, and 78.90% total digestible nutrients (DM basis), along with essential amino acids including methionine, lysine, histidine, valine, and leucine [3, 4]. Studies have reported higher concentrations of iron, zinc, and potassium in *G. bimaculatus* compared to conventional plant and animal sources [5]. Beyond its nutritional applications, *G. bimaculatus* extracts have demonstrated therapeutic potential, effectively counteracting steatosis and apoptosis, while also mitigating intestinal permeability to microbial toxins and suppressing cellular inflammation [6].

One of the major cost components of cricket production is insect feed [7], which has driven interest in developing artificial diets incorporating agricultural by-products. Given that crickets are omnivorous species, they would be expected to digest a wide range of plant- and animal-derived matter [1]. However, studies on diets formulated with agro-industrial byproducts and co-products have shown that plant fiber levels exceeding 10% negatively impact growth and feed conversion efficiency in this species [8–11]. A common factor among these studies is that they were conducted with crickets reared in captivity and maintained on synthetic diets for extended periods, suggesting that the intestinal microbiota responsible for facilitating plant fiber digestion may be reduced or absent.

To support microbiome-based strategies for improving cricket farming, we pursued two complementary objectives. First, we characterized and compared the gut bacterial communities of wild and farmed *G. bimaculatu*s using high-throughput sequencing of the 16 S rRNA gene and functionally screened culturable isolates for traits relevant to insect nutrition. Second, we hypothesized that enhancing the functional capacity of the gut microbiota through probiotic supplementation could improve fiber digestion and rearing performance. To test this, we selected a host-associated bacterial isolate with broad enzymatic capabilities and evaluated its probiotic potential in vivo, using a high-fiber diet formulated from agricultural by-products.

## Materials and Methods

### Sampling of Wild and Farmed Gryllus Bimaculatus Populations

Between 3 and 4 wild adult crickets were collected from each of four different locations in Spain (Castelldefels (3 individuals), Aldehuela-Salamanca (3), St. Guim de Freixenet (3), and Rota, Andalucía (4)) during the last week of September 2022 (Supplementary Table 1). Wild individuals were tracked at night by following their chirps, and upon localization, they were manually captured and placed separately in sterile plastic containers, immediately introduced in a portable cooler containing ice packs, and transported to the laboratory. Adult crickets from production farms were provided by three anonymous private insect breeders based in Spain and the Netherlands, with three individuals per producer. In both cases, upon arrival at the laboratory, insects were immediately stored at -20 °C which also served as the method of euthanasia, until further processing. The insects were transported in plastic containers equipped with 5 × 5 cm egg carton pieces to increase the available surface area, along with the corresponding confidential feed used by each company. To confirm that the collected individuals belonged to *G. bimacualtus*, one hind leg from each insect was removed, and DNA was extracted using the DNeasy Blood and Tissue Kit (Qiagen Sciences), following the manufacturer’s protocol. The mitochondrial cytochrome oxidase I (COI) gene was amplified using the primers LCO1490 (5′-GGTCAACAAATCATAAAGATATTGG-3′) and HCO2198 (5′-TAAACTTCAGGGTGACCAAAAAATCA-3′), targeting a 708 bp fragment. PCR products were sequenced using the Sanger method, and the resulting sequences were compared against the NCBI nucleotide database (GenBank) for species identification.

### Bacterial DNA Extraction and Sequencing

After euthanizing the insects, the whole insect underwent a three-step surface sterilization protocol: brief immersion in sterile water, followed by 70% ethanol treatment, and a final sterile water rinse. Then the complete digestive tract was extracted under sterile conditions and homogenized in 2 ml of 0.9% NaCl solution. Half of the homogenate was reserved for DNA extraction, while the remainder underwent serial dilutions for bacterial isolation.

For microbial community assessment between wild and farmed crickets, individual guts samples were processed separately. Microbial DNA was isolated from 1mL of gut homogenate using DNeasy Blood and Tissue kit protocols (Qiagen Sciences). The processed DNA samples were forwarded to Novogen (UK) for library construction (NEB Next^®^ Ultra™ II FS DNA PCR-free Library Prep Kit, New England Biolabs, USA) and sequencing. The bacterial community was profiled by targeting the V3-V4 variable region of 16 S rRNA using primers 341 F (CCTAYGGGRBGCASCAG) and 806R (GGACTACNNGGGTATCTAAT) on an Illumina NextSeq platform with 250 bp paired-end reads. Sequencing libraries were generated using following manufacturer’s recommendations and indexes were added. The 16 S rRNA (V3–V4) metabarcoding amplicon sequences have been deposited in NCBI GenBank under accession number PRJNA1271293.

### Bioinformatic Analyses

Amplicon sequence processing and downstream diversity analyses were performed following the same workflow described in Saati-Santamaría et al. [12]. Briefly, sequence data were processed in QIIME2 (v2023.2) [13]. Following demultiplexing and quality control using q2-demux, sequences were clustered into amplicon sequence variants (ASVs) via DADA2 [14]. Taxonomic assignment utilized SILVA reference database (version 138.1) [15]. We used rarefaction curves to estimate the adequacy of sequencing depth and the representativeness of the samples. Diversity analyses included both alpha metrics (Shannon, Pielou evenness, Faith’s phylogenetic diversity) and beta metrics (Bray-Curtis and UniFrac distances). To test significant differences in community composition between groups, a PERMANOVA (Adonis test) [16, 17] was applied using the beta-group-significance method. Finally, ANCOM-BC [18] was employed to determine differentially abundant taxa between groups.

### Isolation of the Gut Bacterial Symbionts with Probiotic Potential

Serial dilutions from each gut sample were plated on Luria Bertoni (LB) (Sigma-Aldrich) agar medium (1.5%) and incubated at 28 °C for 72 h. LB was selected as a non-selective complex medium to maximize the recovery of culturable heterotrophic bacteria from the gut, consistent with its widespread use in insect gut microbiome studies [19–22]. Colonies displaying distinct morphological characteristics were isolated, purified through sequential subculturing, and preserved in glycerol at -80 °C for long-term storage.

To assess plant polymer degradation capabilities, bacterial isolates were cultured on LB agar (1.5%) supplemented with specific substrates: 0.5% carboxymethyl cellulose (CMC) or birchwood xylan to evaluate cellulolytic and xylanolytic activity, respectively; and 1% potato starch or citrus peel pectin to test for amylolytic and pectinolytic activity, respectively (all substrates from Sigma-Aldrich, Darmstadt, Germany) [23]. Enzymatic activity was detected by the formation of clear halos around bacterial colonies following staining with 1% Congo red (for cellulose and xylan degradation) or Lugol’s iodine solution (for starch and pectin degradation).

The isolates were also evaluated for their ability to metabolize nitrogen compounds. Uric acid degradation was assessed following the methodology of Callegari et al. [24], by observing the formation of clear halos around isolates spotted onto Nutrient Broth (NB) (Sigma-Aldrich)–Uric Acid plates (0.8% NB, 0.5% uric acid, and 1.5% agar) after incubation at 28 °C for 48 h. Urease activity was determined using urea broth (Fluka Biochemika) in 96-well plates incubated at 28 °C for 48 h without agitation. A color change in the medium from yellow to pink indicated positive urease activity.

### Identification of the Gut Bacterial Symbionts

For probiotic potential identification, only bacteria with the ability to hydrolyze all the plant polymers and metabolize the nitrogen compounds were identified. For that, bacterial DNA was extracted using DNeasy Blood and Tissue Kit (Qiagen Sciences) according to manufacturer guidelines. The 16 S rRNA gene amplification used universal primers 27 F (AGAGTTTGATCATGGCTCAG) and 1492R (TACGGTTACCTTGTTACGACTT). PCR reactions (25 µL) contained 3 µL template DNA, 15 µL REDTaq^®^ ReadyMix™ (Merck), 2.5 µL of each primer (2 µM), and 2 µL ddH2O. The PCR protocol consisted of initial denaturation (95 °C, 9 min), followed by 35 cycles of 94 °C for 60 s, 56.2 °C for 90 s, and 72 °C for 90 s, with final extension at 72 °C for 7 min. After purification with GeneJET PCR Purification Kit (Thermo Fisher Scientific), products were sequenced by Nucleus Services (Salamanca, Spain). The resulting sequences were aligned using BioEdit [25] and compared to type strains’ sequences in the EzTaxon 16 S rRNA [26] database through the EzBioCloud platform [27].

### Genome Sequencing and Analysis

The bacterial strain I53BLB was cultured overnight in liquid LB medium at 28 °C with shaking at 200 rpm. Cells were harvested by centrifugation at 6,000 × g relative centrifugal force (RCF), the supernatant was discarded, and the cell pellet was resuspended in 500 µL of 0.9% NaCl. Preserved cells were shipped to Plasmidsaurus (USA) service for genomic DNA extraction and whole-genome sequencing.

Genomic DNA extraction, library preparation, sequencing, and primary bioinformatic processing were performed by Plasmidsaurus following their standard protocols using the Zymo Quick-DNA Fungal/Bacteria Miniprep Kit. Whole-genome sequencing was carried out using a hybrid Oxford Nanopore Technologies (ONT) and Illumina approach. Amplification-free ONT long-read sequencing libraries were prepared using v14 library preparation chemistry with minimal, sequence-independent fragmentation via tagmentation, and sequenced using R10.4.1 flow cells with a primer-free protocol. Basecalling was performed using Dorado (Oxford Nanopore Technologies [28]) in Super-Accurate mode with default Q10 quality filtering.

ONT reads were filtered by removing the lowest-quality 5% of reads using Filtlong v0.2.1 [29]. Genome size estimation and read downsampling were performed using Autocycler helper functions, and multiple de novo assemblies were generated using Flye v2.9.6+ [30], Hifiasm [31], and Plassembler v1.8.0+ [32]. Assemblies were subsequently processed with Autocycler [33] to remove low-coverage and small contigs and to identify the best consensus assembly, which was rotated to an optimal start position using dnaapler. The long-read assembly was polished using Medaka v1.8.0.For hybrid polishing, paired-end Illumina short reads (2 × 150 bp) were generated and used exclusively to further polish the ONT assembly using Polypolish v0.6.0 [34]. Genome annotation was performed using Bakta v1.11. Assembly quality, completeness, and contamination were assessed using CheckM v1.2.2. The final hybrid assembly achieved an estimated consensus accuracy of approximately Q60 (≥ 99.9999%). with an assembled coverage of 110x. The whole genome sequence and 16 S ribosomal RNA sequence of *P. agglomerans* I53BLB have been deposited in NCBI GenBank under accession numbers PRJNA1404461 and PV702186.1, respectively. Species-level taxonomic assignment of strain I53BLB was assessed using genome-wide similarity metrics. Average Nucleotide Identity based on BLAST (ANIb) was calculated using pyANI [35], comparing the I53BLB genome against reference genomes within the genus *Pantoea*. Digital DNA–DNA hybridization (dDDH) values were computed using the Type (Strain) Genome Server (TYGS) with default parameters [36]. Species assignment was confirmed based on established thresholds for bacterial species delineation (ANI ≥ 95% and dDDH ≥ 70%) [37, 38]. Plasmid replication type, mobility potential, and conjugation systems were predicted using MOB-suite (specifically mob_typer v3.1.9), which classifies plasmids based on relaxase type, mating pair formation (MPF) systems, and predicted mobilization capacity [39]. Plasmids were categorized as non-mobilizable or conjugative according to the presence or absence of complete conjugation machinery. Additional confirmation of plasmid identity was performed using PlasmidHunter [40].

Genome annotation was performed using bakta (v1.11.14) [41], which predicts protein-coding sequences, rRNAs, tRNAs, and other genomic features using curated bacterial databases. Functional annotation of predicted proteins was refined using KofamKOALA, assigning KEGG Orthology (KO) identifiers based on profile hidden Markov models and adaptive score thresholds [42]. Genes involved in uric acid degradation and nitrogen recycling were identified based on these annotations.

Carbohydrate-active enzymes (CAZymes) were identified using dbCAN3, integrating HMMER, DIAMOND, and Hotpep approaches [43]. Only CAZyme predictions supported by at least two methods were retained to ensure annotation confidence. Glycoside hydrolases (GHs) were classified into families and subfamilies according to the CAZy database, and their genomic localization (chromosomal versus plasmid-borne) was determined based on contig assignment.

### High-Fiber Experimental Diet Preparation

An experimental diet was formulated using locally sourced agrifood co-products and by-products with high fiber content, specifically dry bread remains, wheat bran (Biobram, Alcobendas, Madrid), and maize distillers dried grains with solubles (DDGS) (COPASA, Salamanca, Spain), organic Soybean meal (Biobram, Alcobendas, Madrid) was also incorporated to match the crude protein content of the control diet. The crude fiber content of each ingredient was obtained from the technical data sheets provided by the suppliers, with the exception of dry bread remains, for which crude fiber was determined by proximal analysis (< 2%). The final nutritional composition of the experimental diet was as follows: crude protein 17.5%; fat 5.13%; crude fiber 23%; energy 2450 Kcal/kg. All ingredients were ground and passed through a 0.7 mm sieve to obtain a homogeneous flour-based presentation. The control diet consisted of ground organic chicken feed (0.7 mm) (Piensos Ecológicos BIBE, Pajares de la Laguna, Salamanca) containing wheat, maize, soybean meal, sunflower seed cake, powdered beans and soy oil (crude protein: 17.5%; fat: 5.2%; crude fiber: 5%; energy: 2500 Kcal/kg). Both diets were formulated to be isoproteic (17.5% crude protein) but differed in fiber content and were not fully isoenergetic (2450 vs. 2500 Kcal/kg). Both diets were supplemented with an identical amount of a mineral premix. It should be noted that all ingredients were sourced from commercial products with standardized technical data sheets, intentionally chosen for their local availability. However, as no proximal analysis of the final mixed diet was performed, variation in ingredient composition depending on processing should be considered a limitation when replicating this formulation.

### Probiotic Cells Separation

A bacterial isolate capable of hydrolyzing four plant polymers and metabolizing nitrogen compounds was selected and identified as *Pantoea agglomerans* I53BLB. For its potential probiotic application, the strain was cultured in two 2-L flasks, each containing 500 mL of LB liquid medium, at 28 °C for 48 h at 180 rpm, following inoculum preparation protocols reported for *P. agglomerans* strains in comparable studies [39]. One of the flasks was then autoclaved at 121 °C for 15 min for bacterial inactivation, a method previously validated for obtaining postbiotic/heat-inactivated bacterial controls in insect feeding experiments . Cells were obtained by centrifuging the culture at 6,000 × g RCF for 7 min, followed by three washes with 0.9% NaCl. Serial dilutions were performed to achieve an optical density (OD) of 1 (600 nm wavelength). This procedure was applied to both live and heat-inactivated cells.

### Evaluation of a High-Fiber Diet Supplemented with P. Agglomerans

A feeding experiment was conducted to evaluate the impact of *P. agglomerans* I53BLB on cricket performance, using the bacteria as both a probiotic supplement and a hydration source. Two diet types were tested: organic chicken feed (the conventional diet routinely provided to crickets) and the experimental high-fiber diet. Within each diet type, three probiotic supplementation conditions were evaluated: no probiotic (water only), viable *P. agglomerans*, or heat-inactivated *P. agglomerans*, resulting in six experimental groups: (i) organic chicken feed with water (C + W), serving as the control for the standard diet; (ii) organic chicken feed with viable *P. agglomerans* (C + LP); (iii) organic chicken feed with heat-inactivated *P. agglomerans* (C + AP); (iv) experimental diet with water (ED + W), serving as the control for the high-fiber diet; (v) experimental diet with viable *P. agglomerans* (ED + LP); and (vi) experimental diet with heat-inactivated *P. agglomerans* (ED + AP). The probiotic suspension (1 × 10⁸ CFU/mL) replaced water in the probiotic treatments, simultaneously providing hydration and probiotic supplementation. This concentration was selected as an intermediate dose within the range reported in probiotic studies in insects, where effective doses have ranged from 1 × 10⁶ to 1 × 10¹¹ CFU/mL [44–46].

Each experimental unit consisted of a 4-L plastic container (14 × 26 × 19 cm) with five 0.3-cm ventilation holes. Inside each container, two 50-mm Petri dishes provided the diet (organic chicken feed or experimental diet) and the hydration/probiotic source, while two 15 × 15 cm egg cartons increased the available surface area. Each treatment included ten replicates with ten two-week-old nymphs per replicate, supplied with ad libitum feed and 5 mL of the assigned hydration/probiotic source, replenished every two days. All containers were maintained at 29 °C, 60% RH, and a 12-h photoperiod. After three weeks, wet biomass, survival rate, and the percentage of individuals reaching adulthood were recorded.

Subsequently, to evaluate whether the treatments affected progeny output, a subset of 40 individuals per treatment (5 males and 5 females per replicate, with four replicates per treatment) was selected from those that had reached adulthood. These adults were placed in new containers under the same environmental conditions and allowed to mate. Egg hatching was monitored over an 8-day period, with egg counts conducted every 48 h at four consecutive time points. Although hatching was tracked at each interval, data analysis was based on the total number of hatched eggs accumulated over the entire 8-day period for each replicate.

### Histological Analysis of the Insect Midgut

Following completion of the high-fiber diet supplemented with *P. agglomerans* evaluation, two adult individuals (one male and one female) were randomly selected from each treatment group (W + ED and LP + ED) were cold-anesthetized at -20 °C for 2 min prior to sacrifice. The digestive system was subsequently dissected, and midgut tissues were isolated for histological examination.

Middle midgut samples were fixed in 2.5% glutaraldehyde for 2 h, followed by four successive washes in phosphate buffer (30 min each). Tissues were then post-fixed with osmium tetroxide and dehydrated through a graded ethanol series. Following dehydration, samples were embedded in 8% Epon resin and sectioned into semi-thin sections (~ 6 μm) using a microtome.

Sections were mounted on glass slides using egg albumin solution (~ 1% w/v) as an adhesive and dried for 24 h prior to staining. Slides were stained with toluidine blue and examined under light microscopy for morphological analysis.

### Statistical Analysis of the Feeding Experiment

Data normality was assessed using Shapiro-Wilk tests. As several variables did not meet the assumptions of normality, non-parametric statistical methods were applied. The effects of diet type (control versus experimental), probiotic treatment (water, live probiotic, and heat-inactivated probiotic), and their interaction were evaluated using Kruskal-Wallis rank sum tests. For the interaction analysis, we examined all six treatment combinations simultaneously. When significant differences were detected, post-hoc pairwise comparisons were performed using Dunn’s test with Bonferroni adjustment for multiple comparisons. The analyses included average cricket wet weight, survival rate, percentage of individuals reaching the adult stage, and the total number of hatched eggs. All statistical analyses were performed in R version 4.0 [47], with a significance threshold of *p* = 0.05. Data distributions across treatment groups were visualized using box plots generated with the ggplot2 package (version 3.5.2) [48].

## Results

### Gut Bacteriome Diversity and Composition of Wild and Farmed G. Bimaculatus

A total of 1,567,041 high-quality reads (16 S rRNA gene) were obtained from 22 gut samples of *G. bimaculatus*, with an average of 71,229 ± 14,303 reads per sample. These sequences were classified into 5,062 unique amplicon sequence variants (ASVs). Rarefaction analysis based on observed features showed that sequencing depth was adequate to capture most of the bacterial diversity across samples (see Supplementary Figure S1A).

**Fig. 1:**
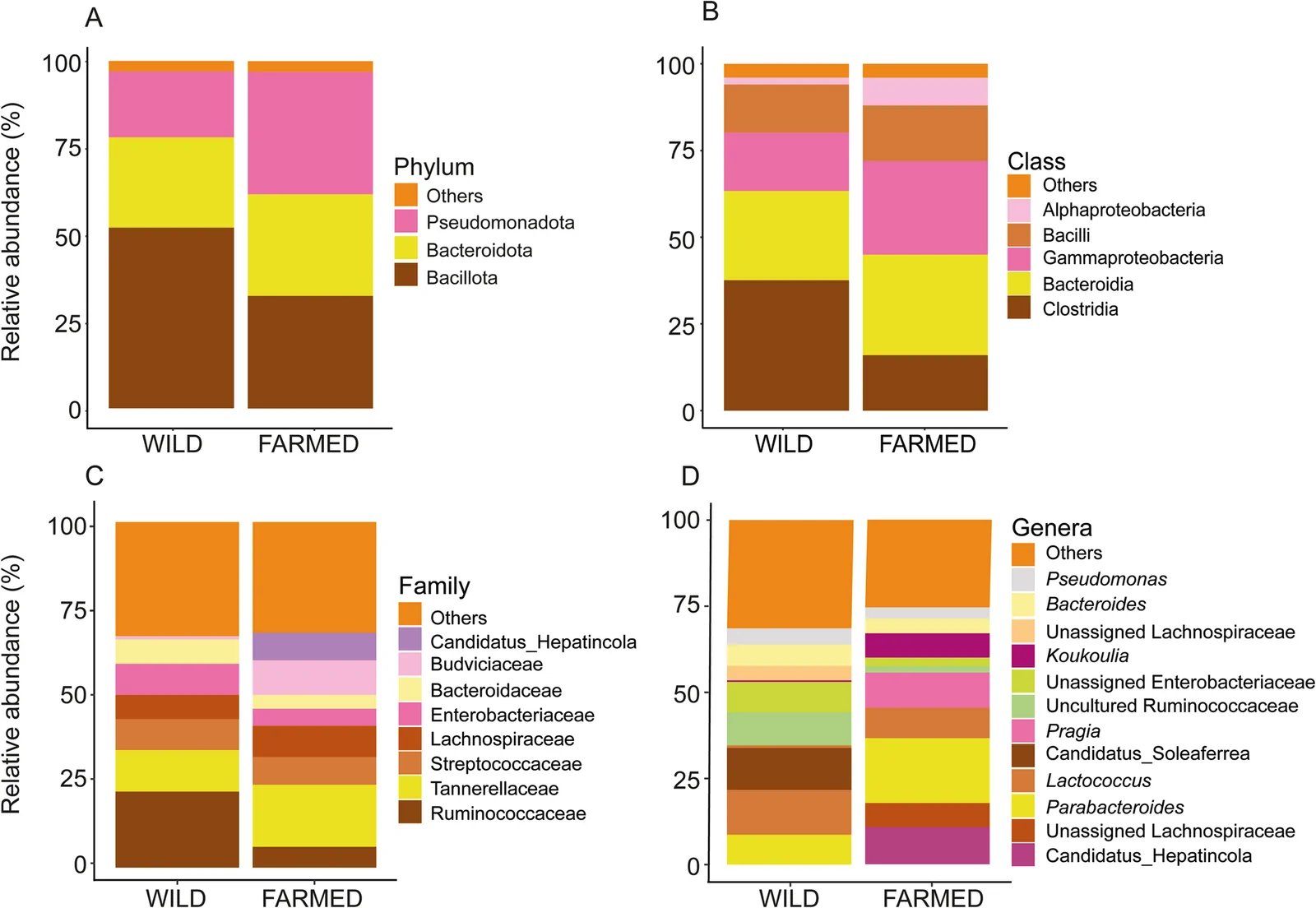
Relative abundance of bacterial composition in the gut of wild and farmed G. bimaculatus based on 16 S rRNA gene analysis. A phylum, B class, C family and D genera levels

Taxonomic classification revealed a total of 24 phyla, 36 classes, 94 orders, 182 families, and 379 genera across the gut microbiota of the 22 samples analyzed. The bacteriome of the gut wild crickets were dominated by the phyla *Bacillota* (52%), *Bacteroidetes* (26%) and *Pseudomonadota* (19%), while the main phyla of the bacteriome of the gut of farmed crickets were *Pseudomonadota* (35%), *Bacillota* (32%) and *Bacteroidetes* (29%) (Fig. 1A). The gut of wild crickets was dominated by the class *Clostridia* (38%), *Bacteroidia* (26%), *Gammaproteobacteria* (17%) and Bacilli (14%). In contrast, the most abundant class in the gut of farmed crickets were *Bacteroidia* (29%), *Gammaproteobacteria* (27%), Clostridia (16%) and Bacilli (16%) (Fig. 1B). At the genus level, in wild *G. bimaculatus*, Candidatus_Soleaferrea (11%), *Parabacteroides* (9%), and *Lactococcus* (9%) were most common, while farmed crickets had *Parabacteroides* (17%), *Candidatus_Hepatincola* (10%), and *Pragia* (9%) (Fig. 1D; Supplementary Figure S1B).

**Fig. 2:**
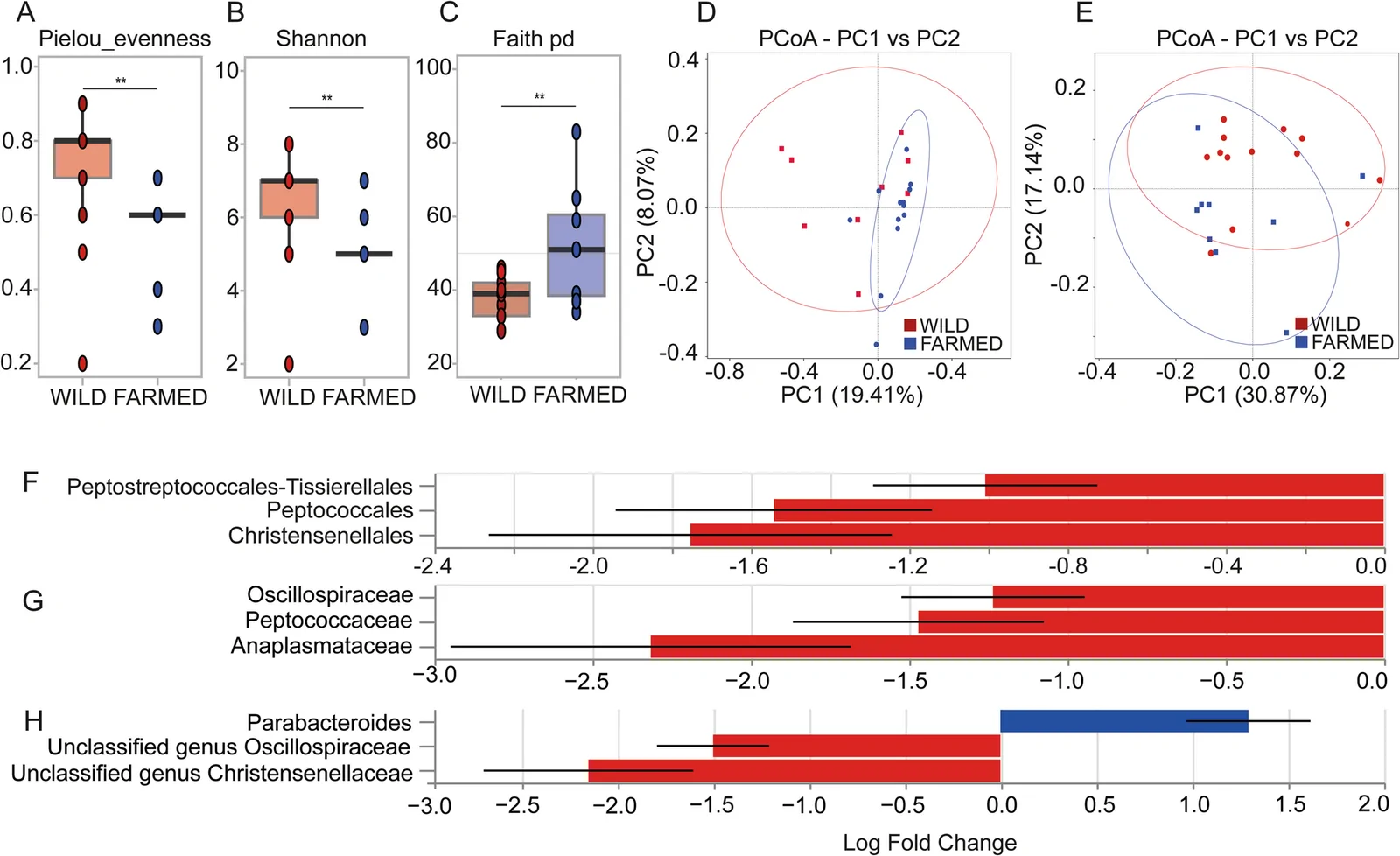
Microbial diversity and composition analysis between wild and farmed *G. bimaculatus*. Alpha diversity indices are shown through boxplots for **A** Pielou’s Evenness, **B** Shannon, and **C** Faith’s PD. ** *p* < 0.01. Beta diversity is shown through principal coordinate analysis (PCoA) plots of **D** Bray-Curtis weighted and **E** unweighted UniFrac distances. Red dots represent wild samples, and blue dots represent farmed samples. Analysis of composition of microbiomes (ANCOM-BC) showing differences in gut bacterial **F** orders, **G** families, and **H** genera between wild and farmed populations. Bar plots represent the log fold change in relative abundances of bacterial taxa, with blue bars indicating enrichment in the farmed population and red bars indicating enrichment in the wild population

Although both farmed and wild crickets harbored diverse gut bacterial communities, the Shannon index was consistently higher in wild crickets (Kruskal-Wallis test: H = 12.79 with *p* < 0.01). In contrast, Faith’s Phylogenetic Diversity revealed that the gut of farmed crickets contained a higher phylogenetic diversity compared to the wild counterparts (H = 12.4 with *p* < 0.01). However, the results of Pielou’s evenness index suggested a more uneven distribution, with certain taxa such as *Parabacteroides*, *Candidatus_Hepatincola*, and *Pragia* being more dominant in abundance in the farmed crickets compared to the wild crickets (H = 5.0 with *p* < 0.01) (Fig. 2A, B and C).

We found that group (wild vs. farmed) significantly explained variation in microbial community composition (ADONIS, *R*² = 0.09, *p* < 0.001). Subsequent pairwise comparisons based on Bray-Curtis, weighted UniFrac, and unweighted UniFrac distances confirmed significant differences between wild and farmed crickets (pairwise PERMANOVA, *p* < 0.001), with clear separation along the axes (Fig. 2D–F).

We identified several taxonomic groups that showed different abundances between the gut bacteriome of wild and farmed crickets. At the genus level, the unassigned genera of the Oscillospiraceae and Christensenellaceae families were more abundant in the guts of wild crickets (LFC = -1.0 and − 0.8 respectively; *p-adj* < 0.05), while the genus *Parabacteroides* was more abundant in the guts of farmed crickets (LFC = + 1.8; *p-adj* < 0.05) (Fig. 2F-H).

### Identification of the core Bacterial Genera in the gut of G. Bimaculatus

Characterizing the core microbiota, defined as the set of microbial taxa consistently present across most individuals is essential to understand the stable and potentially functional components of the gut bacterial community. These core members are likely to play key roles in host physiology, digestion, immunity, or overall microbiome resilience, and may help distinguish between host-driven and environment-driven variation. In our dataset, 15 genera were identified as a core bacteria, being present in over 90% of the samples. Among the most abundant taxa in the core microbiome relative to the entire bacterial community were *Lactococcus* (5.1% mean relative abundance), *Bacteroides* (0.4%), *Wolbachia* (0.33%), and *Enterococcu*s (0.32%).

Eight genera were shared across the core microbiota of wild and farmed crickets, including *Bacteroides*, *Bifidobacterium*, *Candidatus Soleaferrea*,* Desulfovibrio*,* Enterococcus*,* Lachnoclostridium*,* Lactobacillus*, and *Lactococcus*. In wild crickets, 16 genera were identified as core members, with *Lactococcus* (4.7%), *Bacteroides* (0.47%), *Wolbachia* (0.45%), and *Enterococcus* (0.29%) being the most abundant. In contrast, farmed crickets harbored 12 core genera, among which *Lactococcus* (3.6%), *Parabacteroides* (0.50%), *Lachnoclostridium* (0.23%), and *Bacteroides* (0.17%) were the most abundant.

### Isolation and Identification of the gut Bacterial Symbionts

**Fig. 3:**
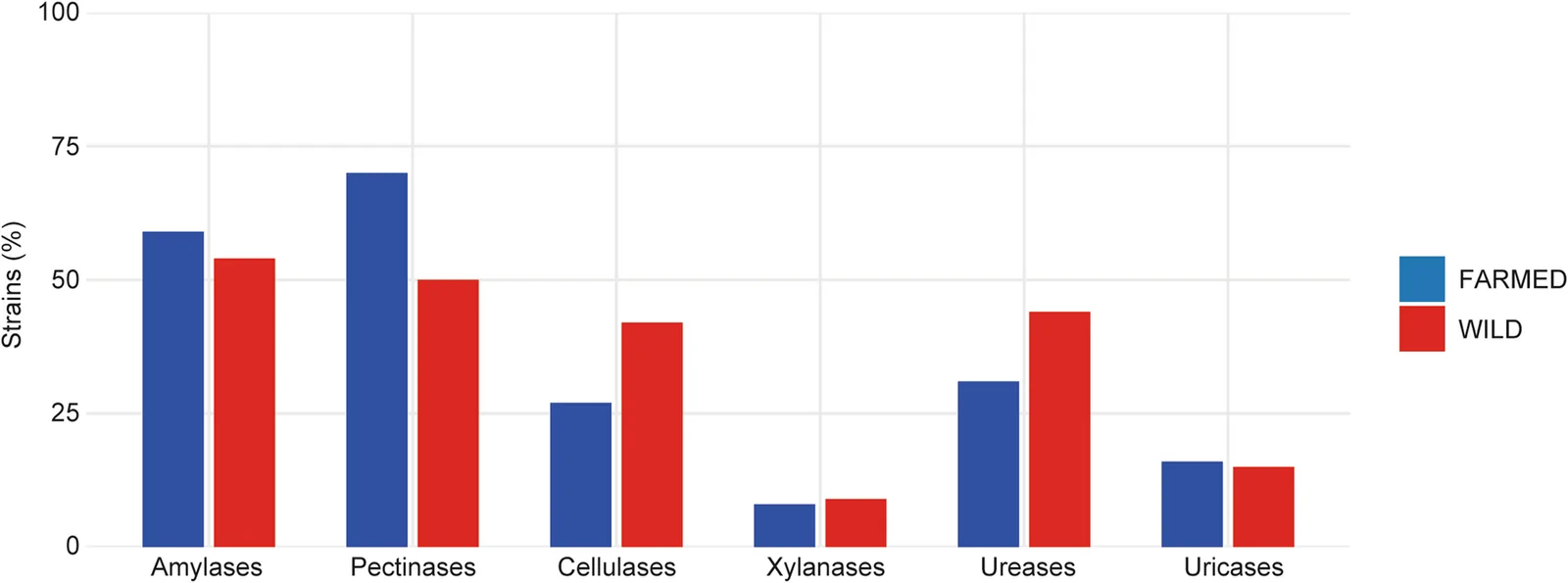
Percentage of bacterial isolates from the gut of *G. bimaculatus* exhibiting enzymatic activities associated with polysaccharide degradation and nitrogen metabolism. Red bars correspond to wild individuals, while blue bars represent farmed individuals

**Fig. 4:**
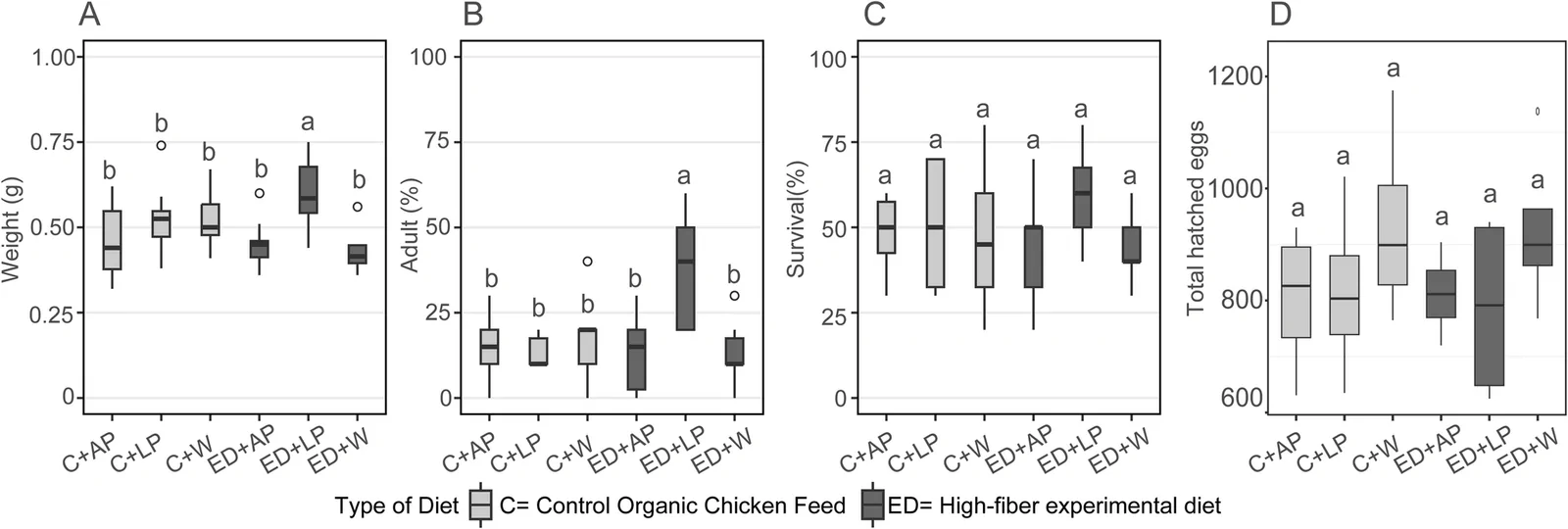
Box plots representing a full factorial analysis of the effects of diet type and probiotic supplementation on **A** individual weight (g), **B** percentage of individuals reaching the adult stage, **C** survival percentage, and **D** total number of hatched eggs in *G. bimaculatus*. “C” and “ED” represent the control organic chicken feed and high-fiber experimental diet, respectively. “AP” represents autoclaved (heat-inactivated) *P. agglomerans* (I53BLB), “LP” represents live *P. agglomerans* (I53BLB), and “W” represents water control treatments. Different lowercase letters indicate significant differences between treatment combinations (*p* < 0.05)

A total of 199 pure cultures were isolated from the gut of *G. bimaculatus*, 134 originated from wild specimens and 65 from farmed crickets. Among the wild cricket isolates, 54%, 50%, 42%, and 9% demonstrated the ability to hydrolyze starch, pectin, cellulose, and xylan, respectively (Fig. 4A). Two isolates, identified as *P. agglomerans* I53BLB, and *Bacillus cereus* I173B (100% similarity to the type strain) could degrade all four compounds simultaneously. Additionally, 44% and 15% of our isolates hydrolyzed uric acid and urea, respectively (Fig. 3).In farmed *G. bimaculatus*, 59%, 70%, 27%, and 8% of isolates were able to hydrolyze pectin, starch, cellulose, and xylan, respectively (Fig. 3). One strain was capable of degrading all four polysaccharides and was initially assigned to the genus *Klebsiella* based on EzBioCloud analysis; however, the 16 S rRNA gene sequence shared only 94.25% similarity with *Klebsiella huaxiensis*, the closest validly named taxon. This level of identity suggests that these isolates may represent a novel genus within the family *Enterobacteriaceae*. Additionally, 31% and 16% hydrolyzed uric acid and urea, respectively (Fig. 3).

### Genomic Features Related to Polysaccharide Degradation and Nitrogen Recycling in Pantoea Agglomerans I53BLB

We focused on *Pantoea agglomerans* I53BLB due to its unique combination of traits identified during our isolation and screening process. *P. agglomerans* I53BLB was selected for probiotic evaluation because it demonstrated the ability to hydrolyze all four plant polymers tested, as well as uric acid. Although other isolated bacterial species, such as *Bacillus cereus*, showed similar metabolic capabilities, this species has been frequently reported as a pathogen in various insect species and may also pose a food safety risk [49, 50]. In addition, *Pantoea* was present in 80% of the sampled individuals, suggesting a possible prior adaptation of this microorganism to the digestive system of *G. bimaculatus*. These features make *P. agglomerans* I53BLB a prime candidate for exploring the genetic basis of multifunctional symbiosis and its probiotic potential.

The genome of *Pantoea agglomerans* I53BLB consists of a single chromosome and three fully closed plasmids, with an estimated total size of approximately 4.96 Mb (GC = 55.13%). The three plasmids vary in size: contig_2 is 551 kb, contig_3 is 173 kb, and contig_4 is 156 kb. Based on replication and mobility predictions, contigs 2 and 3 are non-mobilizable, while contig 4 is conjugative, suggesting potential for horizontal gene transfer. Taxonomic assignment based on genomic analyses confirmed that I53BLB belongs to the species *Pantoea agglomerans*, with a digital DNA-DNA hybridization (dDDH) value of 79.4% and an average nucleotide identity (ANIb) of 97.6% relative to the type strain *Pantoea agglomerans* DSM 3493 (GCA_019048385.1).

*Pantoea agglomerans* I53BLB harbors a diverse arsenal of glycoside hydrolases (GHs) enabling degradation of major plant polysaccharides including pectin, starch, cellulose, and xylan. Chromosomally encoded primary pectin degradation is supported by GH28 endo-polygalacturonases and GH105 rhamnogalacturonan lyases, while secondary activities involve GH43 arabinofuranosidases and GH2 β-galactosidases. Starch degradation relies on multiple GH13 subfamilies (GH13_5, GH13_10, GH13_18) acting at the primary level, supplemented by secondary α-glucosidases (GH31) and β-glucosidases (GH1). Cellulose breakdown is mainly enabled by GH8 endoglucanases and β-glucosidases (GH3, GH1), facilitating the sequential hydrolysis of cellulose polymers. Xylan degradation appears limited to secondary enzymes such as β-glucosidases (GH1) and β-galactosidases (GH2_10), indicating partial utilization of hemicellulosic substrates.

Interestingly, several GH genes related to these degradative pathways are also localized on plasmids, notably β-glucosidases and arabinogalactan endo-β-1,4-galactosidase (GH53), which may confer adaptive advantages through horizontal gene transfer. This versatile enzymatic repertoire supports *P. agglomerans* I53BLB’s role in polysaccharide turnover within the insect gut, contributing to host nutrition and microbiome modulation. The combined chromosomal and plasmid-borne GH genes highlight metabolic plasticity, underpinning its efficacy as a probiotic capable of decomposing complex dietary polymers.

In addition to carbohydrate metabolism, the chromosome encodes a complete set of genes involved in uric acid degradation and nitrogen recycling. Key enzymes such as urate oxidase (*uaZ*), FAD-dependent urate hydroxylases (*hpxO*, *hpyO*), and 5-hydroxyisourate hydrolases (*uraH* – 2 copies in the chromosome and another in a plasmid–, *hiuH*) are present, alongside allantoinase (*allB*), allantoate deiminase (*allC*), and downstream enzymes responsible for conversion of ureidoglycine to oxalurate and ultimately urea and oxalate (e.g., *pucG*, *hpxY*, *hpxW*). This genomic configuration suggests a robust capacity to recycle nitrogenous waste compounds within the insect gut environment, potentially enhancing nitrogen availability for both the microbiome and the host. The presence of these genes on plasmids further implies potential horizontal gene transfer and adaptive flexibility.

Together, the enzymatic profiles for polysaccharide degradation and nitrogen recycling emphasize the ecological role of *P. agglomerans* I53BLB as a multifunctional symbiont, capable of improving host nutrient utilization from complex and nitrogen-rich substrates.

### Evaluation of a High-Fiber Diet Supplemented with P. Agglomerans I53BLB

Plant high-fiber diets are usually avoided in cricket farming as they have been related to decreased feed conversion ratios and reduced survival rates [51, 52]. However, current knowledge indicates that crickets function as non-specialized omnivores, displaying preferences for plant material along with other food sources [53]. We hypothesize that *P. agglomerans* I53BLB could help crickets tolerate such high-fiber intake when used as a probiotic. Therefore, we also included this variable in our experimental set-up.

We found that the average weight of individuals differed significantly across probiotic treatments (Kruskal-Wallis χ² = 11.45, d.f. = 2, *p* = 0.0033), but no significant differences were observed between diet types (χ² = 0.47, d.f. = 1, *p* = 0.4914). However, the interaction between diet and probiotic treatment had a significant effect on weight (χ² = 18.8, d.f. = 5, *p* = 0.0021). Post hoc comparisons showed that within the Experimental diet (ED), individuals receiving the live probiotic (LP) treatment had significantly higher weights (mean = 0.602 g) than those receiving the Autoclaved probiotic (AP) (mean = 0.451 g, *p* = 0.0351) or water (mean = 0.427 g, *p* = 0.0027) treatments (Fig. 4A). In contrast, under the C diet (Control organic Chicken Feed), no significant differences in weight were found between probiotic treatments (all *p* > 0.05), although median weights were slightly higher in the LP group (Fig. 4A). Overall, the LP strain was associated with the greatest weight gain, particularly under the ED diet.

Regarding the percentage of individuals reaching the adult stage, no significant differences were detected between diet types alone (χ² = 1.87, d.f. = 1, *p* = 0.1717) or among probiotic treatments alone (χ² = 5.06, d.f. = 2, *p* = 0.0797). However, the interaction between diet and probiotic treatment had a significant effect (χ² = 17.7, d.f. = 5, *p* = 0.0033). Post hoc Dunn’s tests revealed that ED + LP treatment resulted in significantly higher adult percentages (mean = 37%) compared to C + LP (mean = 13%, *p* = 0.008), ED + AP (mean = 13%, *p* = 0.016), and ED + W (mean = 13%, *p* = 0.007) treatments (Fig. 4B).

Survival percentage did not significantly differ across diet types (χ² = 0.004, d.f. = 1, *p* = 0.952) or probiotic treatments (χ² = 4.31, d.f. = 2, *p* = 0.116), and the interaction between these factors was also not significant (χ² = 6.66, d.f. = 5, *p* = 0.247) (Fig. 4C).

Additionally, no significant differences were found in the number of hatched eggs among diet types (χ² = 0.23, d.f. = 1, *p* = 0.631) or probiotic treatments (χ² = 1.65, d.f. = 2, *p* = 0.437). Likewise, the combination of diet and probiotic treatment did not significantly affect egg hatching (χ² = 4.08, d.f. = 5, *p* = 0.538) (Fig. 4D).

### Histological Analysis of the Effects of P. Agglomerans I53BLB on the Middle Midgut of G. Bimaculatus

**Fig. 5:**
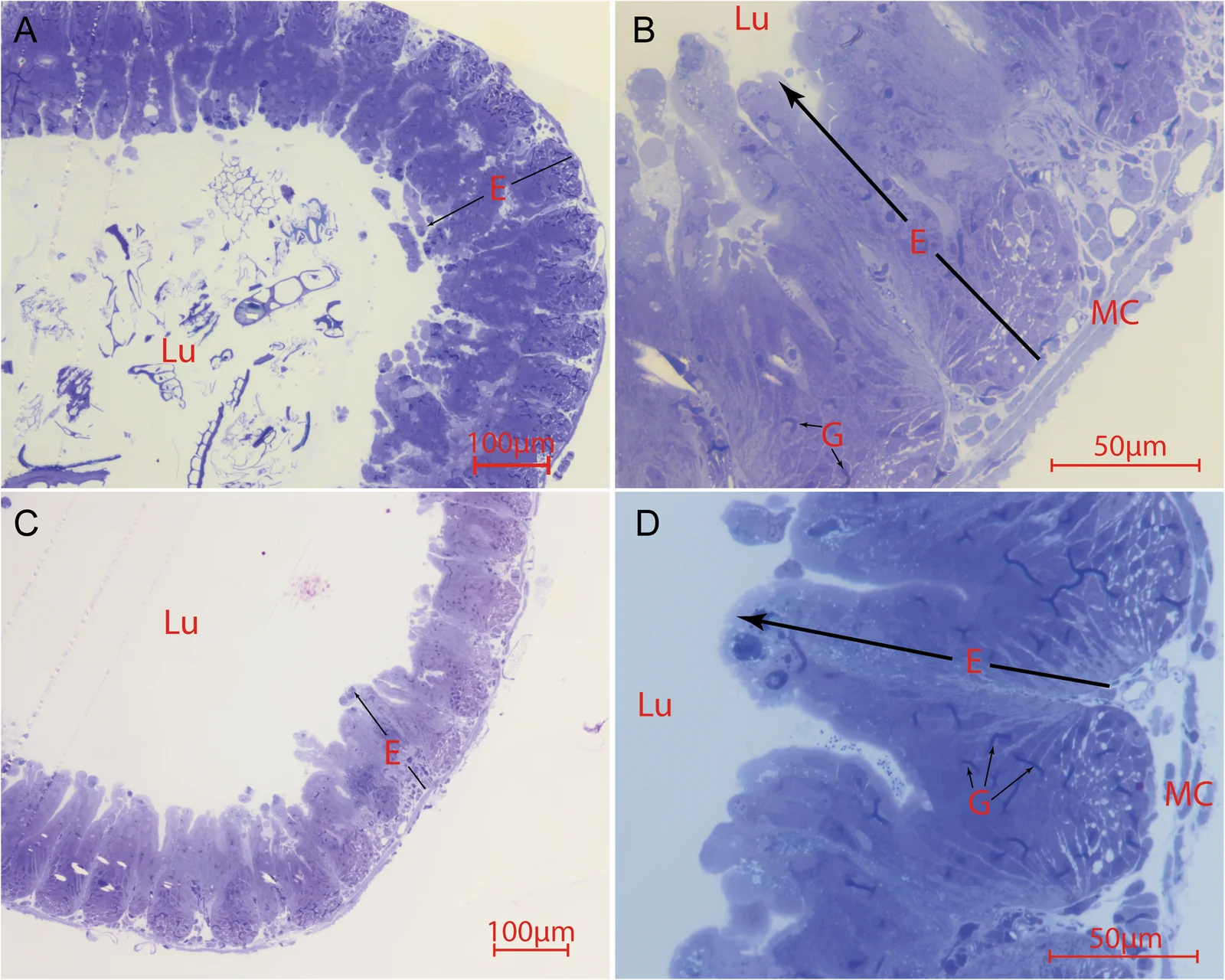
Midgut histology in treatment groups. Cross-sections of middle midgut stained with toluidine blue showing (**A**, **B**) W + ED (experimental diet + water) and (**C**, **D**) LP + ED (live probiotic + experimental diet) treatments. Normal epithelial architecture was observed in both groups. Lu, lumen; E, epithelium; G, glandular cells; MC, muscle cells: A, C = 100 μm; B, D = 50 μm

Histological examination of the middle midgut cross-sections revealed no observable differences in tissue morphology between treatment groups. Both ED + W (experimental diet + water) and ED + LP (experimental diet + live probiotic) exhibited normal epithelial architecture with well-preserved cellular organization (Fig. 5). The epithelial layer showed uniform arrangement with intact basement membrane and proper cellular differentiation. Glandular cells maintained their characteristic morphology and distribution pattern, while muscle cells displayed normal structural organization. No signs of tissue damage, cellular degeneration, or inflammatory responses were detected in either treatment group. The lumen remained clear without cellular debris or abnormal accumulations. These findings indicate that *P. agglomerans* I53BLB supplementation did not induce detectable histopathological changes in the middle midgut of *G. bimaculatus* under the experimental conditions tested.

## Discussion

Probiotics are increasingly being recognized as promising tools to support health and performance in insect farming systems [54, 55]. Despite the growing interest in insect–microbe symbioses, this study is the first to evaluate the probiotic potential of a host-associated bacterium in crickets of industrial relevance, specifically in *G. bimaculatus*, a species of high commercial importance in the edible insect industry. As part of our research, we compared the gut microbiota of wild and farmed crickets, identifying notable differences in microbial composition. We then isolated and characterized bacterial strains with functional traits potentially beneficial to the host, selecting candidates for in vivo testing. Our results demonstrate that supplementing a high-fiber diet with a *P. agglomerans* I53BLB, originally isolated from the gut of *G. bimaculatus*, led to measurable improvements in growth performance. These findings highlight the potential of microbiome-informed interventions in cricket farming and open new avenues for enhancing productivity and sustainability in the edible insect sector.

### Gut Bacteriome Diversity and Composition of Wild and Sarmed G. Bimaculatus

We found a clear difference in the gut bacteriome between wild and farmed populations. In *G. bimaculatus*, wild crickets showed higher Shannon diversity but lower phylogenetic faith diversity than their farmed counterparts. This intriguing pattern suggests that while wild crickets harbour a greater diversity of bacterial species, farmed crickets have a more phylogenetically diverse bacterial community, probably due to the exposure to human-associated bacteria in captivity [56]. The lower Pielou evenness in farmed crickets indicates a more uneven distribution of bacterial taxa, possibly due to selection pressure in the farmed cricket environment. This is more in line with the general trend observed in other animals, where wild populations tend to have a more diverse gut microbiome. These results are consistent with previous studies on other insect species, such as the cricket *Teleogryllys oceanicus* [57], the stag beetle *Dorcus hopei hopei* [58] and the silkworm *Bombyx mori* [59], where domestication led to a shift in the diversity of the gut microbiome.

This shift may be related to differences in diet composition between wild and farmed crickets. *Pseudomonadota* have been associated with herbivorous diets in Ensifera [60], which aligns with the synthetic, plant-based feed typically provided to farmed crickets, such as commercial chicken feed composed of easily assimilated ingredients. *Bacteroidota*, in turn, have been reported as one of the most abundant phyla in captive animals including insects, particularly in animals fed highly processed or chemically defined diets [57, 59, 61, 62]. In crickets, the increased *Bacteroidota* abundance has been linked to diets rich in simple carbohydrates, including those lacking fats [63], as well as high-protein/low-carbohydrate compositions [64].

Additionally, we found that orders *Peptostreptococcales-Tissierellales*,* Peptococcales*, and *Christensenellales*, and the families *Oscillospiraceae*, *Peptococcaceae*, and *Anaplasmataceae* were highly enriched in wild populations compared to their farmed counterparts. *Peptostreptococcales-Tissierellales* order is commonly found in the digestive tract and feces of various animal species, including insects, it remains poorly studied due to its strict anaerobic nature. Members of this group are typically detected in environments rich in decomposing organic matter, anaerobic sediments, and industrial digesters [65, 66]. Functionally, *Peptostreptococcales-Tissierellales* primarily participates in fermentative processes, utilizing substrates such as amino acids, simple sugars, or organic acids to produce metabolites like short-chain fatty acids and methylated compounds. Additionally, this order has been identified as part of microbial consortia capable of degrading lignocellulose, contributing redundant functionality to the decomposition of plant biomass [67].

The elevated abundance of *Peptococcales*,* Christensenellales*, and their corresponding families *Peptococcaceae*,* Christensenellaceae*, along with *Oscillospiraceae* in wild crickets, compared to their farmed counterparts, may offer nutritional and physiological advantages. These bacterial groups are well known for their ability to ferment complex, non-digestible carbohydrates [68–70], potentially enabling wild crickets to extract energy from otherwise inaccessible dietary components. Although this has not yet been thoroughly investigated in insects, studies in other animal species have shown that the fermentation carried out by these microbial taxa leads to the production of short-chain fatty acids (SCFAs), which have been associated with reduced gastrointestinal pH, thereby limiting pathogen growth, and enhanced mineral absorption [71, 72].

The higher abundance of genera *Parabacteroides* in farmed crickets aligns with previous studies on cricket gut microbiomes. This genus has been consistently identified as a core component of the microbiome of the *Gryllus assimilis* and *Acheta domesticus* [73]. The prevalence of these anaerobic bacteria across different cricket species suggests a conserved functional role in the cricket gut. *Parabacteroides* are known to be involved in the fermentation of plant polysaccharides, which is crucial for the digestive processes in the cricket hindgut (Kaufman and Klug, 1991). The higher abundance of *Parabacteroides* in farmed crickets may reflect adaptations to the specific dietary composition in captivity, potentially rich in plant-based ingredients that are easily fermentable. Interestingly, these genera have also been observed in cricket frass and cricket feed, suggesting a possible transmission through the food chain and its environment [73].

Most core bacterial genera were present at low abundance, except *Lactococcus*; specifically, *Lactococcus garvieae* was consistently detected at high relative abundance in the amplicon-based taxonomic profiles of the gut microbiome (Supplementary Table 2). *L. garvieae* has been observed in gut ecosystems across multiple orthopteran species, suggesting functional significance; for instance, in *Locusta migratoria*, this bacterium constituted a predominant taxon throughout developmental stages and farmed and wild populations indicating potential roles in development and nutritional processes [24, 74]. Also, this species is associated with effective cellulose degradation, enhancing the host’s capacity to metabolize plant-derived diets containing fibrous components [74]. Research on *Chorthippus parallelus* identified *L. garvieae* within the core microbiome in both gut and reproductive tissues, suggesting reproductive functions [75]. In *Anabrus simplex* and *Scapsipedus marginatus* this bacterium aids nutrient acquisition and predominates in gut regions involved in carbohydrate metabolism and immunity [62, 76]. The consistent presence of this bacterium across orthopteran species and developmental stages indicates contributions to crucial physiological mechanisms.

### Gut bacterial Symbionts with Probiotic Potential

The metabolic potential of culturable gut-associated bacteria isolated from *G. bimaculatus* differed notably between wild and farmed individuals, reflecting potential adaptations to their respective environments and diets. Wild crickets harbored a higher proportion of isolates capable of degrading cellulose and hemicelluloses, plant polysaccharides commonly found in natural vegetation. This aligns with the broader and less controlled diet of wild insects, which likely includes fibrous plant materials requiring specialized microbial enzymes for digestion. In contrast, farmed crickets showed higher percentages of pectin and starch-hydrolyzing bacteria, suggesting an enrichment of microbial taxa adapted to simpler carbohydrates, likely due to the inclusion of fruits, vegetables, and processed feeds in their diet which is common in farmed crickets [77]. Fruits and vegetables are rich in pectin, while feeds such as chicken mash which contain corn meals is a starch-rich feed component [78], together promoting the growth of pectinolytic and amylolytic bacteria. Despite the differences in carbohydrate-degrading profiles, both environments supported a small subset of bacterial strains capable of hydrolyzing all four plant-derived polymers. Interestingly, these strains belonged to distinct taxa in each group: *P. agglomerans* (I53BLB) in wild crickets, and potentially novel taxa within the family *Enterobacteriaceae* in farmed individuals. Beyond their ecological relevance, these metabolic traits also point to potential biotechnological applications, as gut-associated bacteria from other herbivorous insects have been shown to harbor lignocellulolytic enzymes of interest for biomass conversion, bioremediation, and industrial processes [23].

Wild *G. bimaculatus* harbored a higher proportion of and uricolytic (44%) bacterial isolates compared to farmed individuals (31%). This enrichment likely reflects the nitrogen limitations of their natural habitats, where the insect diet typically low in bioavailable nitrogen is complemented by mutualistic microbes. Simultaneously, uricolytic bacteria help recycle nitrogen by breaking down uric acid, a major nitrogenous waste in insects, into ammonia and other usable forms [79]. These processes can support insect development under protein-restricted conditions. Additionally, wild crickets foraging behavior may expose them to rhizosphere-associated microbes present in the soil and plant surfaces. Many rhizosphere bacteria possess functional traits related to nitrogen cycling, such as nitrogen fixation and mineralization [80]. Since they use burrows as shelters and oviposition sites, frequent contact with the rhizosphere may facilitate the acquisition of these beneficial symbionts [81].

### Evaluation of a High-Fiber Diet Supplemented with P. Agglomerans I53BLB

The significant interaction between diet and probiotic treatment on both body weight and adult emergence percentage suggests that the beneficial effects of *P. agglomerans* are diet-dependent rather than occurring across all dietary conditions. This finding suggests that the *P. agglomerans* I53BLB strain may possess unique properties that promote host growth only when combined with the experimental diet. Several studies have reported that *P. agglomerans* is a common symbiont in the digestive systems of insects, providing various physiological advantages such as improved digestion, nitrogen acquisition, and enhanced survival [82–84], contributing to nitrogen and phosphate availability, detoxification of plant allelochemicals, suppression of entomopathogens by disrupting their microbial partners, and synthesis of bioactive molecules such as pheromones and antifungal metabolites [51, 85, 86]. In line with this, *P. agglomerans* have also been predominantly associated with the digestive system of *L. migratoria*, particularly in wild populations where it forms part of the core bacteriome of this species, as well as in *Lissorhoptrus oryzophilus*. This consistent association reinforces the idea that its primary function is related to nutrient assimilation and digestive efficiency [24, 86]. Similarly, Jin and Kim, [87] demonstrated that feeding *Frankliniella occidentalis* with ampicillin significantly reduced bacterial load and delayed development, but supplementation with *P. agglomerans* isolate BFoK1 effectively rescued the developmental delay, supporting the role of *P. agglomerans* as beneficial symbionts in insect development. Beyond insects, even lipopolysaccharides (LPS) derived from *P. agglomerans* have demonstrated beneficial effects across other animal models, including improved growth performance, enhanced immune activity, and increased intestinal goblet cell density in rainbow trout (*Oncorhynchus mykiss*) [88], and immune stimulation with tumor remission in human cancer patients [89]. These multifunctional roles support its potential as a beneficial probiotic, particularly in enhancing host metabolism, immunity, and development.

From an industrial perspective, the increase in the percentage of crickets reaching adulthood within the same time frame could translate into shorter production cycles, improved turnover, and reduced feeding. Considering that feed represents one of the most substantial expenses in cricket production, often accounting for 60–70% of the total operating cost [90, 91], any reduction in time to harvest can significantly enhance economic efficiency. This rationale aligns with findings from aquaculture and poultry systems, where small reductions in production cycles are associated with considerable financial gains. For instance, Luna et al., [92] demonstrated that optimizing harvest timing and production strategies in aquaculture can increase profits by up to 40%, and Szőllősi et al., [93] reported that reducing the broiler production cycle by just one day may raise net income by approximately €0.55/m² annually.

Significant interactions between diet and probiotic treatment were observed for both body weight and adult emergence, suggesting synergistic effects between the high-fiber diet and the probiotic. The enhanced performance in the LP group may result from *P. agglomerans* I53BLB capacity to degrade plant polysaccharides [94], thereby making more nutrients available to the host. Given that the experimental diet contained approximately five times more fiber than the control, it is plausible that its indigestible components acted as prebiotic substrates, fostering a gut environment that favored the colonization and metabolic activity of *P. agglomerans* I53BLB. In contrast, in the control diet, composed of more readily digestible ingredients, this additional polysaccharide-degrading functionality would be less relevant, which could explain the smaller effect observed in this group. Notably, part of this polysaccharide-degrading capacity is encoded on plasmids, including glycoside hydrolases, which may further enhance metabolic flexibility and contribute to the diet-dependent probiotic effect observed. In *Pantoea*, large plasmids have been shown to undergo domestication and vertical inheritance, providing stable, specialized functions that contribute to ecological adaptation and niche differentiation, and are known to act as reservoirs of accessory metabolic traits, including specialized biosynthetic functions [12, 95].

The combination of probiotics and prebiotics, known as symbiotics, can enhance insect health and performance by promoting beneficial bacterial activity, including improved fermentation processes, enhanced nutrient availability, and overall modulation of the gut microbiome [55]. In this context, the improved performance observed in the LP group may reflect a symbiotic interaction, where the high-fiber content selectively stimulated the growth or activity of the probiotic strain, ultimately benefiting the host.

Despite the observed weight and adult emergence differences, survival rates and egg hatching did not differ significantly across groups. This may suggest that the benefits conferred by the probiotic are more related to nutrient assimilation and energy allocation rather than to direct effects on mortality resistance or reproductive output. These results are consistent with previous studies in *Bactrocera cucurbitae*,* B. mori*,* Hermetia illucens*, and *Tenebrio molitor*, where probiotics improved growth without significantly affecting survival or reproduction [96–99].

In our study, we did not determine whether *P. agglomerans* I53BLB was transmitted to eggs or whether subsequent generations maintained higher *P. agglomerans* abundance in their gut microbiota with continued probiotic effects. This would be valuable to evaluate in future research, as vertical transmission could make probiotic application more economical by eliminating the need for application in each production cycle if the beneficial effects persist across generations without reapplication.

Interestingly, although *P. agglomerans* I53BLB did not enhance reproductive parameters in this study, this species has been reported to exhibit vertical transmission and has been detected not only in the insect gut but also in reproductive organs such as ovaries [100, 101]. This suggests a potential role in early developmental processes or transgenerational effects. Research by Itoh et al., [102] demonstrate that related *Pantoea* species in stinkbugs like *Dolycoris baccarum* are transmitted vertically via egg-smearing, where specialized enlarged crypts at the posterior end of the midgut in female bugs facilitate symbiont transfer to the egg surface. This transmission mechanism ensures that offspring acquire the symbiont immediately after hatching. Similar mechanisms could potentially be involved with *P. agglomerans* in our study species, which would support the possibility of sustainable, transgenerational probiotic effects and warrants further investigation.

An aspect worth highlighting is that the high-fiber experimental diet (ED) demonstrated comparable performance to the control organic chicken feed (C), with no significant differences in average weight, survival percentage, adult emergence rate, or egg hatching. These results indicate that *G. bimaculatus* could effectively utilize high-fiber diets without compromising performance, at least with the specific ingredients incorporated in our experimental formulation. While our experimental diet contained 23% fiber, and previous research has suggested that fiber content above 20% DM typically reduces cricket growth due to indigestible lignin, cellulose, and hemicellulose components [10, 103, 104], our findings challenge this assumption. This suggests that fiber digestibility may depend significantly on its source rather than merely its quantity.

Our results align with [1], who found that a cassava-based diet with 19% fiber yielded optimal performance in terms of weight gain and survival when properly balanced with protein sources. However, our study did not evaluate the nutritional composition of the resulting crickets. Although these high-fiber diets produced crickets with developmental parameters similar to those reared on commercial diets, determining their protein and fat content remains essential, as one of the primary goals of cricket farming is protein production. Future research should investigate how high-fiber diets affect the nutritional profile of *G. bimaculatus* to further optimize their value as a sustainable protein source.

## Conclusions

This study provides new insights into the microbiome of *G. bimaculatus* and highlights the potential of leveraging host-associated symbionts to improve insect farming efficiency. The comparative analysis of wild and farmed crickets revealed clear differences in gut bacterial composition, with wild individuals harboring a more functionally diverse bacterial community, particularly enriched in fiber-degrading and nitrogen-metabolizing taxa. These findings suggest that prolonged captivity and artificial diets may reduce the presence of beneficial symbionts crucial for digesting complex plant substrates.

Among the isolates characterized, *P. agglomerans* I53BLB stood out for its ability to hydrolyze multiple plant polymers and degrade uric acid. When used as a probiotic in a high-fiber diet, *P. agglomerans* I53BLB, significantly improved weight gain and adult emergence in *G. bimaculatus*, with no negative effects on survival, development, or reproduction. These results indicate a potential symbiotic synergy between this bacterium and the host, especially under dietary conditions that mimic agro-industrial by-product feeding strategies. The performance of the high-fiber diet alone, comparable to that of a commercial control diet, further supports the feasibility of using cost-effective, sustainable ingredients in cricket production.

From an applied perspective, the integration of *P. agglomerans* I53BLB into cricket farming systems offers a promising approach to enhancing growth efficiency and promoting sustainable production. Its high prevalence in wild crickets, combined with broad metabolic capacity, makes it a compelling candidate for probiotic development. Future research should explore optimal dosage and delivery methods, assess persistence across life stages and generations, and evaluate potential for use in other cricket species or insect models. In parallel, a techno-economic assessment should be conducted to determine the cost-benefit balance of implementing this probiotic on an industrial scale. Altogether, these findings underscore the value of microbiome-informed strategies in edible insect farming and pave the way toward more resilient, efficient, and circular production systems.

## Supplementary Information

Below is the link to the electronic supplementary material.

Supplementary figure 1 (PNG 51.6 KB)

High Resolution Image

## Data Availability

Whole genome sequence and 16 S ribosomal RNA sequence of *P. agglomerans* I53BLB have been deposited in NCBI GenBank under accession number PRJNA1404461 and PV702186.1 respectively. The 16 S rRNA (V3–V4) metabarcoding amplicon sequences have been deposited in NCBI under accession numbers PRJNA1271293.

## References

[1] Kwon W, Lee KP (2024) Macronutrient regulation in nymphs of the two-spotted cricket, *Gryllus bimaculatus* (Orthoptera: Gryllidae). J Insect Physiol 157:104684. 10.1016/j.jinsphys.2024.104684 39074715 10.1016/j.jinsphys.2024.104684

[2] Mitchaothai J, Grabowski NT, Lertpatarakomol R, Trairatapiwan T, Chhay T, Keo S, Lukkananukool A (2022) Production Performance and Nutrient Conversion Efficiency of Field Cricket (Gryllus bimaculatus) in Mass-Rearing Conditions. Animals 12:2263. 10.3390/ani12172263 36077983 10.3390/ani12172263 PMC9454574

[3] Murugu DK, Onyango AN, Ndiritu AK, Osuga IM, Xavier C, Nakimbugwe D, Tanga CM (2021) From Farm to Fork: Crickets as Alternative Source of Protein, Minerals, and Vitamins. Front Nutr 8:704002. 10.3389/fnut.2021.704002 34447775 10.3389/fnut.2021.704002 PMC8382788

[4] Phesatcha B, Phesatcha K, Viennaxay B, Matra M, Totakul P, Wanapat M (2022) Cricket Meal (*Gryllus bimaculatus*) as a Protein Supplement on In Vitro Fermentation Characteristics and Methane Mitigation. Insects 13:129. 10.3390/insects13020129 35206703 10.3390/insects13020129 PMC8877429

[5] Magara HJO, Niassy S, Ayieko MA, Mukundamago M, Egonyu JP, Tanga CM, Kimathi EK, Ongere JO, Fiaboe KKM, Hugel S, Orinda MA, Roos N, Ekesi S (2021) Edible Crickets (Orthoptera) Around the World: Distribution, Nutritional Value, and Other Benefits-A Review. Front Nutr 7:537915. 10.3389/fnut.2020.537915 33511150 10.3389/fnut.2020.537915 PMC7835793

[6] Hwang BB, Chang MH, Lee JH, Heo W, Kim JK, Pan JH, Kim YJ, Kim JH (2019) The Edible Insect Gryllus bimaculatus Protects against Gut-Derived Inflammatory Responses and Liver Damage in Mice after Acute Alcohol Exposure. Nutrients 11:857. 10.3390/nu11040857 30995745 10.3390/nu11040857 PMC6521266

[7] Halloran A, Roos N, Hanboonsong Y (2016) Cricket farming as a livelihood strategy in Thailand. Geogr J 183:112–124. 10.1111/geoj.12184

[8] Akiyama D, Kaewplik T, Fujisawa T, Kurosu T, Sasaki Y (2023) Crickets (*Gryllus bimaculatus*) using food waste usefulness of self-selection feed design method through each growth stage. J Insects Food Feed 10:247–258. 10.1163/23524588-20230077

[9] Dobermann D, Michaelson L, Field LM (2019) The effect of an initial high-quality feeding regime on the survival of *Gryllus bimaculatus* (black cricket) on bio-waste. J Insects Food Feed 5:117–124. 10.3920/JIFF2018.0024

[10] Orinda MA, Mosi RO, Ayieko MA, Amimo FA (2017) Growth performance of common house cricket (*Acheta domesticus*) and field cricket (*Gryllus bimaculatus*) crickets fed on agro-byproducts. J Entomol Zool Stud 5:1664–1668

[11] Vo L, Nguyen H, Bui P (2022) Effect of replacing dietary protein with cassava leaf meal on performance and nutritional composition of two-spotted crickets (*Gryllus bimaculatus*). Livest Res Rural Dev 34:1–6

[12] Saati-Santamaría Z (2023) Global map of specialized metabolites encoded in prokaryotic plasmids. Microbiol Spectr 11:e01523–e01523. 10.1128/spectrum.01523-23 37310275 10.1128/spectrum.01523-23 PMC10434180

[13] Bolyen E, Rideout JR, Dillon MR, Bokulich NA, Abnet CC, Al-Ghalith GA, Alexander H, Alm EJ, Arumugam M, Asnicar F, Bai Y, Bisanz JE, Bittinger K, Brejnrod A, Brislawn CJ, Brown CT, Callahan BJ, Caraballo-Rodríguez AM, Chase J, Cope EK, Caporaso JG (2019) Reproducible, interactive, scalable and extensible microbiome data science using QIIME 2. Nat Biotechnol 37:852–857. 10.1038/s41587-019-0209-9 31341288 10.1038/s41587-019-0209-9 PMC7015180

[14] Callahan BJ, McMurdie PJ, Rosen MJ, Han AW, Johnson AJ, Holmes SP (2016) Nat Methods 13:581–583. 10.1038/nmeth.3869 10.1038/nmeth.3869 PMC4927377 27214047

[15] Pruesse E, Quast C, Knittel K, Fuchs BM, Ludwig W, Peplies J, Glöckner FO (2007) SILVA: a comprehensive online resource for quality checked and aligned ribosomal RNA sequence data compatible with ARB. Nucleic Acids Res 35:7188–7196. 10.1093/nar/gkm864 17947321 10.1093/nar/gkm864 PMC2175337

[16] Anderson MJ (2001) A new method for non-parametric multivariate analysis of variance. Austral Ecol 26:32–46. 10.1111/j.1442-9993.2001.01070.pp.x

[17] Oksanen J, Blanchet FG, Friendly M, Kindt R, Legendre P, McGlinn D, Minchin PR, O’Hara RB, Simpson GL, Solymos P, Stevens MHH, Szoecs E, Wagner H (2018) vegan: Community Ecology Package. R package version 2 5-3. https://CRAN.R-project.org/package=vegan

[18] Lin H, Peddada SD (2020) Analysis of compositions of microbiomes with bias correction. Nat Commun 11:3514. 10.1038/s41467-020-17041-7 32665548 10.1038/s41467-020-17041-7 PMC7360769

[19] Cifuentes Y, Vilcinskas A, Kämpfer P, Köhler HR (2022) Isolation of *Hermetia illucens* larvae core gut microbiota by two different cultivation strategies. Antonie Van Leeuwenhoek 115:821–837. 10.1007/s10482-022-01735-7 35460063 10.1007/s10482-022-01735-7 PMC9123031

[20] Dong J, Yao X, Zhang Y, Wu X, Liu X, Zhang H, Jiang H, Hou J, Yan J, Sun J (2025) Gut Bacteria Mediate Aggregation Pheromone Release in the Borer Beetle *Trigonorhinus sp*. Insects 16:999. 10.3390/insects16100999 41148868 10.3390/insects16100999 PMC12564262

[21] Zhang Q, Wang S, Zhang X, Zhang K, Li Y, Yin Y, Zhang R, Zhang Z (2022) Beneficial Bacteria in the Intestines of Housefly Larvae Promote Larval Development and Humoral Phenoloxidase Activity, While Harmful Bacteria do the Opposite. Front Immunol 13:938972. 10.3389/fimmu.2022.938972 35874711 10.3389/fimmu.2022.938972 PMC9299419

[22] Zhang N, He J, Shen X, Sun C, Muhammad A, Shao Y (2021) Contribution of sample processing to gut microbiome analysis in the model Lepidoptera, silkworm Bombyx mori. Comput Struct Biotechnol J 19:4658–4668. 10.1016/j.csbj.2021.08.020 34504661 10.1016/j.csbj.2021.08.020 PMC8390955

[23] Fabryová A, Kostovčík M, Díez-Méndez A, Jiménez-Gómez A, Celador-Lera L, Saati-Santamaría Z, García-Fraile P (2018) On the bright side of a forest pest – the metabolic potential of bark beetles’ bacterial associates. Sci Total Environ 619:9–17. 10.1016/j.scitotenv.2017.11.074 29136536 10.1016/j.scitotenv.2017.11.074

[24] Callegari M, Jucker C, Fusi M, Leonardi MG, Daffonchio D, Borin S, Savoldelli S, Crotti E (2020) Hydrolytic Profile of the Culturable Gut Bacterial Community Associated With Hermetia illucens. Front Microbiol 11:1965. 10.3389/fmicb.2020.01965 32903451 10.3389/fmicb.2020.01965 PMC7434986

[25] Hall TA (1999) BioEdit: a user-friendly biological sequence alignment editor and analysis program for Windows 95/98/NT. Nucleic Acids Symposium Series 41: 95–98

[26] Kim OS, Cho YJ, Lee K, Yoon SH, Kim M, Na H, Park SC, Jeon YS, Lee JH, Yi H, Won S, Chun J (2012) Introducing EzTaxon-e: a prokaryotic 16S rRNA gene sequence database with phylotypes that represent uncultured species. Int J Syst Evol Microbiol 62(3):716–721. 10.1099/ijs.0.038075-0 22140171 10.1099/ijs.0.038075-0

[27] Chalita M, Kim YO, Park S, Oh HS, Cho JH, Moon J, Baek N, Moon C, Lee K, Yang J, Nam GG, Jung Y, Na SI, Bailey MJ, Chun J (2024) EzBioCloud: a genome-driven database and platform for microbiome identification and discovery. Int J Syst Evol Microbiol 74(6):006421. 10.1099/ijsem.0.006421 38888585 10.1099/ijsem.0.006421 PMC11261700

[28] Oxford Nanopore Technologies (2024) /2025 *Dorado* (Version 1.3.1). [Software]. https://github.com/nanoporetech/dorado

[29] Wick R (2021) Filtlong. github.com/rrwick/Filtlong. Retrieved 14 August 2022

[30] Kolmogorov M, Yuan J, Lin Y, Pevzner P (2019) Assembly of long, error-prone reads using repeat graphs. Nat Biotechnol 37:540–546. 10.1038/s41587-019-0072-8 30936562 10.1038/s41587-019-0072-8

[31] Cheng H, Concepcion GT, Feng X (2021) Haplotype-resolved de novo assembly using phased assembly graphs with hifiasm. Nat Methods 18:170–175. 10.1038/s41592-020-01056-5 33526886 10.1038/s41592-020-01056-5 PMC7961889

[32] Bouras G, Sheppard AE, Mallawaarachchi V, Vreugde S, Holt K (2023) Plassembler: an automated bacterial plasmid assembly tool. Bioinformatics 39(7):btad409. 10.1093/bioinformatics/btad409 37369026 10.1093/bioinformatics/btad409 PMC10326302

[33] Wick R, Howden P, Stinear T (2025) Autocycler: long-read consensus assembly for bacterial genomes. Bioinformatics 41(9):btaf474. 10.1093/bioinformatics/btaf474 40875535 10.1093/bioinformatics/btaf474 PMC12460055

[34] Wick R, Holt E (2022) Polypolish: short-read polishing of long-read bacterial genome assemblies. PLoS Comput Biol 18:e1009802. 10.1371/journal.pcbi.1009802 35073327 10.1371/journal.pcbi.1009802 PMC8812927

[35] Pritchard L, Glover RH, Humphris S, Elphinstone JG, Toth IK (2016) Genomics and taxonomy in diagnostics for food security: soft-rotting enterobacterial plant pathogens. Anal Methods 8:12–24. 10.1039/C5AY02550H

[36] Meier-Kolthoff JP, Göker M (2019) TYGS is an automated high-throughput platform for state-of-the-art genome-based taxonomy. Nat Commun 10:2182. 10.1038/s41467-019-10210-3 31097708 10.1038/s41467-019-10210-3 PMC6522516

[37] Chun J, Oren A, Ventosa A, Christensen H, Arahal DR, da Costa MS, Rooney AP, Yi H, Xu XW, De Meyer S, Trujillo ME (2018) Proposed minimal standards for the use of genome data for the taxonomy of prokaryotes. Int J Syst Evol Microbiol 68:461–466. 10.1099/ijsem.0.002516. 10.1099/ijsem.0.002516 29292687

[38] Saati-Santamaría Z, Flores-Félix JD, Igual JM, Velázquez E, García-Fraile P, Martínez-Molina E (2024) Speciation features of *Ferdinandcohnia quinoae* sp. nov. to adapt to the plant host. J Mol Evol 92:169–180. 10.1007/s00239-024-10164-1 38502221 10.1007/s00239-024-10164-1 PMC10978704

[39] Miech P, Berggen A, Lindberg JE, Chhay T, Khieu B, Jansson A (2016) Growth and survival or reared Cambodian field crickets (*Teleogryllus testaceus*) fed weeds, agricultural and food industry by-products. J Insects Food Feed 2:285–292

[40] Tian R, Zhou J, Imanian B (2024) PlasmidHunter: accurate and fast prediction of plasmid sequences using gene content profile and machine learning. Brief Bioinform 25:bbae322. 10.1093/bib/bbae322 38960405 10.1093/bib/bbae322 PMC11770376

[41] Schwengers O, Jelonek L, Dieckmann MA, Beyvers S, Blom J, Goesmann A (2021) Bakta: rapid and standardized annotation of bacterial genomes via alignment-free sequence identification. Microb Genom 7:e000685. 10.1099/mgen.0.000685 10.1099/mgen.0.000685 PMC8743544 34739369

[42] Aramaki T, Blanc-Mathieu R, Endo H, Ohkubo K, Kanehisa M, Goto S, Ogata H (2020) KofamKOALA: KEGG ortholog assignment based on profile HMM and adaptive score threshold. Bioinformatics 36:2251–2252. 10.1093/bioinformatics/btz859 31742321 10.1093/bioinformatics/btz859 PMC7141845

[43] Zheng J, Ge Q, Yan Y, Zhang X, Huang L, Yin Y (2023) dbCAN3: automated carbohydrate-active enzyme and substrate annotation. Nucleic Acids Res 51:W115–W121. 10.1093/nar/gkad328 37125649 10.1093/nar/gkad328 PMC10320055

[44] Dahal S, Jensen AB, Lecocq A (2022) Effect of Probiotics on Tenebrio molitor Larval Development and Resistance against the Fungal Pathogen Metarhizium brunneum. Insects 13:1114. 10.3390/insects13121114 36555024 10.3390/insects13121114 PMC9788617

[45] Gorrens E, Lecocq A, De Smet J (2023) The Use of Probiotics during Rearing of *Hermetia illucens*: Potential, Caveats, and Knowledge Gaps. Microorganisms 11:245. 10.3390/microorganisms11020245 36838211 10.3390/microorganisms11020245 PMC9960648

[46] Haytham H, Kamel C, Wafa D (2024) Probiotic consortium modulating the gut microbiota composition and function of sterile Mediterranean fruit flies. Sci Rep 14:1058. 10.1038/s41598-023-50679-z 38212383 10.1038/s41598-023-50679-z PMC10784543

[47] R Core Team (2021) R: A language and environment for statistical computing. R Foundation for Statistical Computing, Vienna, Austria. https://www.R-project.org/

[48] Wickham H (2011) ggplot2. Wiley Interdiscip. Rev Comput Stat 3:180–185. 10.1002/wics.147

[49] Elsharkawy MM, Almasoud M, Alsulaiman YM, Baeshen RS, Elshazly H, Kadi RH, Hassan MM, Shawer R (2022) Efficiency of *Bacillus thuringiensis* and *Bacillus cereus* against *Rhynchophorus ferrugineus*. Insects 13:905. 10.3390/insects13100905 36292853 10.3390/insects13100905 PMC9604075

[50] Frentzel H, Kelner-Burgos Y, Fischer J, Heise J, Göhler A, Wichmann-Schauer H (2022) Occurrence of selected bacterial pathogens in insect-based food products and in-depth characterisation of detected *Bacillus cereus* group isolates. Int J Food Microbiol 379:109860. 10.1016/j.ijfoodmicro.2022.109860 35933921 10.1016/j.ijfoodmicro.2022.109860

[51] Ghaderi Jouybari M, Malbobi MA, Rezaei Pour V, Saharkhiz Bandforouzi K, Siadati SA (2009) Characterization of *Pseudomonas putida* (P13) and *Pantoea agglomerans* (P5) as novel probiotic on phosphate availability and performance in broiler. Analele IBNA 25:40–41

[52] Kuo C, Fisher BL (2022) A literature review of the use of weeds and agricultural and food industry by-products to feed farmed crickets (Insecta; Orthoptera; Gryllidae). Front Sustain Food Syst 5:810421. 10.3389/fsufs.2021.810421

[53] Rowe E, Robles López KY, Robinson KM, Baudier KM, Barrett M Farmed cricket (Acheta domesticus, Gryllus assimilis, and Gryllodes sigillatus; Orthoptera) welfare considerations: Recommendations for improving global practice. J. Insects Food Feed 10(8), 1253–1311., Saati-Santamaría Z, Vicentefranqueira R, Kolařik M, Rivas R, García-Fraile P (2024) 2023. Microbiome specificity and fluxes between two distant plant taxa in Iberian forests. Environ. Microbiome 18, 64 10.1163/23524588-00001087 10.1186/s40793-023-00520-x PMC10363313 37481564

[54] Häbermann J, Antunes B, Haase H, Rolff J, Keil C, Rafaluk C (2025) Boosting Bug Farms: A Meta-Analysis on Probiotic Effects in Insect Rearing. bioRxiv. 10.1101/2025.04.04.647199

[55] Savio C, Mugo-Kamiri L, Upfold JK (2022) Bugs in bugs: The role of probiotics and prebiotics in maintenance of health in mass-reared insects. Insects 13:376. 10.3390/insects13040376 35447818 10.3390/insects13040376 PMC9025317

[56] Bensch HM, Lundin D, Tolf C, Waldenström J, Zöttl M (2023) Environmental effects rather than relatedness determine gut microbiome similarity in a social mammal. J Evol Biol 36:1753–1760. 10.1111/jeb.14208 37584218 10.1111/jeb.14208

[57] Ng SH, Stat M, Bunce M, Simmons LW (2018) The influence of diet and environment on the gut microbial community of field crickets. Ecol Evol 8:4704–4720. 10.1002/ece3.3977 29760910 10.1002/ece3.3977 PMC5938447

[58] Lu Y, Chu S, Shi Z, You R, Chen H (2024) Marked variations in diversity and functions of gut microbiota between wild and domestic stag beetle *Dorcus hopei hopei*. BMC Microbiol 24:24. 10.1186/s12866-023-03177-1 38238710 10.1186/s12866-023-03177-1 PMC10795464

[59] Chen B, Du K, Sun C, Vimalanathan A, Liang X, Li Y, Wang B, Lu X, Li L, Shao Y (2018) Gut bacterial and fungal communities of the domesticated silkworm (*Bombyx mori*) and wild mulberry-feeding relatives. ISME J 12:2252–2262. 10.1038/s41396-018-0174-1 29895989 10.1038/s41396-018-0174-1 PMC6092317

[60] Sánchez-Torres V, González-Candelas L, Marcet-Houben M, Gabaldón T, Teixidó N, Viñas I, Usall J (2026) Biological control of postharvest diseases in apple by *Pantoea agglomerans* CPA-2: unravelling its mechanisms of action. Postharvest Biol Technol 234:114174. 10.1016/j.postharvbio.2025.114174

[61] Wang Y, Yang X, Zhang M, Pan H (2023) Comparative Analysis of Gut Microbiota between Wild and Captive Golden Snub-Nosed Monkeys. Animals 13:1625. 10.3390/ani13101625 37238055 10.3390/ani13101625 PMC10215246

[62] Smith C, Srygley B, Healy F, Swaminath K, Mueller G (2017) Spatial structure of the mormon cricket gut microbiome and its predicted contribution to nutrition and immune function. Front Microbiol 8: 801. 10.3389/fmicb.2017.00801 10.3389/fmicb.2017.00801 PMC5427142 28553263

[63] Gupta M, Velayutham P, Elbeshbishy E, Hafez H, Khafipour E, Derakhshani H, Nakhla G (2014) Co-fermentation of glucose, starch, and cellulose for mesophilic biohydrogen production. Int J Hydrogen Energy 39:20958–20967. 10.1016/j.ijhydene.2014.10.079

[64] David LA, Maurice CF, Carmody RN, Gootenberg DB, Button JE, Wolfe BE, Biddinger SB (2014) Diet rapidly and reproducibly alters the human gut microbiome. Nature 505:559–563. 10.1038/nature12820 24336217 10.1038/nature12820 PMC3957428

[65] Devane ML, Taylor W, Dupont PY, Armstrong B, Weaver L, Gilpin BJ (2023) Exploring the Bacterial Community in Aged Fecal Sources from Dairy Cows: Impacts on Fecal Source Tracking. *Microorganisms* 11, 1161. 10.3390/microorganisms11051161 10.3390/microorganisms11051161 PMC10223543 37317135

[66] Yan M, Treu L, Campanaro S, Tian H, Zhu X, Khoshnevisan B, Tsapekos P, Angelidaki I, Fotidis IA (2020) Effect of ammonia on anaerobic digestion of municipal solid waste: Inhibitory performance, bioaugmentation and microbiome functional reconstruction. Chem Eng J 401:126159. 10.1016/j.cej.2020.126159

[67] Ali Q, Ma S, Farooq U, Liu B, Wang Z, Sun H, Cui Y, Li D, Shi Y (2024) Chronological dynamics of the gut microbiome in response to the pasture grazing system in geese. Microbiol Spectr 12:e0418823. 10.1128/spectrum.04188-23 39189756 10.1128/spectrum.04188-23 PMC11448393

[68] Konikoff T, Gophna U (2016) *Oscillospira*: a Central, Enigmatic Component of the Human Gut Microbiota. Trends Microbiol 24:523–524. 10.1016/j.tim.2016.02.015 26996766 10.1016/j.tim.2016.02.015

[69] Qin W, Song P, Lin G, Huang Y, Wang L, Zhou X, Li S, Zhang T (2020) Gut microbiota plasticity influences the adaptability of wild and domestic animals in co-inhabited areas. Front Microbiol 11:125. 10.3389/fmicb.2020.00125 32117147 10.3389/fmicb.2020.00125 PMC7018712

[70] Waters JL, Ley RE (2019) The human gut bacteria *Christensenellaceae* are widespread, heritable, and associated with health. BMC Biol 17:83. 10.1186/s12915-019-0699-4 31660948 10.1186/s12915-019-0699-4 PMC6819567

[71] Gultemirian ML, Corti HR, Perez Chaia A, Apella MC (2014) Fermentation in vitro of a mixture of dietary fibers and cane molasses by the cecal microbiota: Application on mineral absorption through the laying hen’s colonic epithelium. Anim Feed Sci Technol 191:76–82. 10.1016/j.anifeedsci.2014.01.019

[72] Mann ER, Lam YK, Uhlig HH (2024) Short-chain fatty acids: Linking diet, the microbiome and immunity. Nat Rev Immunol 24:577–595. 10.1038/s41577-024-01014-8 38565643 10.1038/s41577-024-01014-8

[73] Aleknavičius D, Lukša J, Strazdaitė-Žielienė Ž, Servienė E (2022) The Bacterial Microbiota of Edible Insects *Acheta domesticus* and *Gryllus assimilis* Revealed by High Content Analysis. Foods 11:1073. 10.3390/foods11081073 35454659 10.3390/foods11081073 PMC9032608

[74] Li K, Li WJ, Liang K, Li FF, Qin GQ, Liu JH, Li XJ (2024) Gut microorganisms of *Locusta migratoria* in various life stages and its possible influence on cellulose digestibility. mSystems. 10.1128/msystems.00600-24. e00600-24 38888356 10.1128/msystems.00600-24 PMC11264664

[75] Martínez-Rodríguez P, Hernández-Pérez M, Bella JL (2013) Detection of *Spiroplasma* and *Wolbachia* in the bacterial gonad community of *Chorthippus parallelus*. Microb Ecol 66:211–223. 10.1007/s00248-013-0226-z 23588850 10.1007/s00248-013-0226-z

[76] Gatheru J (2019) Analysis of Microbial Load and Diversity in Crickets (Gryllus bimaculatus and Scapsipedus icipe) used as a source of protein for food. All Master’s Theses. 18. https://thehive.icipe.org/all-theses/18

[77] Halloran A, Megido C, Oloo J, Weigel T, Nsevolo P, Francis F (2018) Comparative aspects of cricket farming in Thailand, Cambodia, Lao People’s Democratic Republic, Democratic Republic of the Congo and Kenya. J Insects Food Feed 15(2):101–114. 10.3920/JIFF2017.0016

[78] Straub P, Tanga CM, Osuga I, Windisch W, Subramanian S (2019) Experimental feeding studies with crickets and locusts on the use of feed mixtures composed of storable feed materials commonly used in livestock production. Anim Feed Sci Technol 255:114215. 10.1016/j.anifeedsci.2019.114215

[79] Ren X, Guo R, Akami M, Niu C (2022) Nitrogen acquisition strategies mediated by insect symbionts: a review of their mechanisms, methodologies, and case studies. Insects 13:84. 10.3390/insects13010084 35055927 10.3390/insects13010084 PMC8781418

[80] Yang Y, Xu N, Zhang Z, Lei C, Chen B, Qin G, Qiu D, Lu T, Qian H (2024) Deciphering microbial community and nitrogen fixation in the legume rhizosphere. J Agric Food Chem 72(11):4453–4464. 10.1021/acs.jafc.3c07425 10.1021/acs.jafc.3c09160 38442360

[81] Okada J, Mizuta S, Toh Y (2011) Refuge Size Preference in the Field Cricket *Gryllus bimaculatus*. Zool Sci 28:243–248. 10.2108/zsj.28.243 10.2108/zsj.28.243 21466340

[82] El Khoury S, Gauthier J, Mercier PL, Moïse S, Giovenazzo P, Derome N (2024) Honeybee gut bacterial strain improved survival and gut microbiota homeostasis in *Apis mellifera* exposed in vivo to clothianidin. Microbiol Spectr 12:e0057824. 10.1128/spectrum.00578-24 39189755 10.1128/spectrum.00578-24 PMC11448422

[83] Li WH, Jin DC, Li FL, Cheng Y, Jin JX (2017) Metabolic phenomics of bacterium *Pantoea* sp. from larval gut of the diamondback moth, *Plutella xylostella* (Lepidoptera: Plutellidae). Symbiosis 72:135–142. 10.1007/s13199-016-0453-4

[84] Shukla SP, Beran F (2020) Gut microbiota degrades toxic isothiocyanates in a flea beetle pest. Mol Ecol 29:4692–4705. 10.1111/mec.15657 33006166 10.1111/mec.15657

[85] Lorenzi AS, Bonatelli ML, Chia MA, Peressim L, Quecine MC (2022) Opposite sides of Pantoea agglomerans and its associated commercial outlook. Microorganisms 10:2072. 10.3390/microorganisms10102072 36296348 10.3390/microorganisms10102072 PMC9610544

[86] Zhang J, Huang Y, Huang X, Jiang M (2016) Infection state of *Pantoea agglomerans* in the rice water weevil *Lissorhoptrus oryzophilus* (Coleoptera: Curculionidae). Appl Entomol Zool 51:561–569. 10.1007/s13355-016-0433-4

[87] Jin G, Kim Y (2023) *Pantoea* bacteria isolated from three thrips (*Frankliniella occidentalis*, *Frankliniella intonsa*, and *Thrips tabaci*) in Korea and their symbiotic roles in host insect development. J Microbiol Biotechnol 33:745–752. 10.4014/jmb.2301.01018 36994621 10.4014/jmb.2301.01018 PMC10331939

[88] Skalli A, Castillo M, Andree KB, Tort L, Furones D, Gisbert E (2013) The LPS derived from the cell walls of the Gram-negative bacteria *Pantoea agglomerans* stimulates growth and immune status of rainbow trout (*Oncorhynchus mykiss*) juveniles. Aquaculture 416–417:272–279. 10.1016/j.aquaculture.2013.09.037

[89] Morishima A, Inagawa H (2016) Clinical Effects of Orally Administered Lipopolysaccharide Derived from *Pantoea agglomerans* on Malignant Tumors. Anticancer Res 36:3747–3751 27354649

[90] Hanboonsong Y, Durst PB (2020) Guidance on sustainable cricket farming: A practical manual for farmers and inspectors. FAO. https://coilink.org/20.500.12592/w72t3d

[91] Harsányi E, Juhász C, Kovács E, Huzsvai L, Pintér R, Fekete G, Gyuricza C (2020) Evaluation of organic wastes as substrates for rearing *Zophobas morio*, *Tenebrio molitor*, and *Acheta domesticus* larvae as alternative feed supplements. Insects 11:604. 10.3390/insects11090604 32899592 10.3390/insects11090604 PMC7564407

[92] Luna M, Llorente I, Cobo Á (2020) Aquaculture production optimisation in multi-cage farms subject to commercial and operational constraints. Biosyst Eng 194:67–78. 10.1016/j.biosystemseng.2020.05.012

[93] Szőllősi L, Szűcs I, Nabradi A (2014) Economic issues of broiler production length. Ekon Poljoprivreda 61:633–646. 10.5937/ekoPolj1403633S

[94] Patakova P, Vasylkivska M, Sedlar K, Jureckova K, Bezdicek M, Lovecka P, Branska B, Kastanek P, Krofta K (2024) Whole genome sequencing and characterization of *Pantoea agglomerans* DBM 3797, endophyte, isolated from fresh hop (*Humulus lupulus* L). Front Microbiol 15:1305338. 10.3389/fmicb.2024.1305338 38389535 10.3389/fmicb.2024.1305338 PMC10882544

[95] Romero Picazo D, Muccino F, Kwasigroch P, Hartmann L, Hülter NF, Dagan T (2025) Evolution of the plant-associated Pantoea was accompanied by plasmid domestication events. Mol Biol Evol 42(11): msaf273. 10.1093/molbev/msaf273 10.1093/molbev/msaf273 PMC12612815 41159561

[96] Lecocq A, Natsopoulou ME, Berggreen IE, Eilenberg J, Heckmann LHL, Nielsen HV, Stensvold CR, Jensen AB (2021) Probiotic properties of an indigenous *Pediococcus pentosaceus* strain on *Tenebrio molitor* larval growth and survival. J Insects Food Feed 7:309–317. 10.3920/JIFF2020.0156

[97] Pei Y, Zhao S, Chen X, Zhang J, Ni H, Sun M, Lin H, Liu X, Chen H, Yang S (2022) *Bacillus velezensis* EEAM 10B Strengthens Nutrient Metabolic Process in Black Soldier Fly Larvae (*Hermetia illucens*) via Changing Gut Microbiome and Metabolic Pathways. Front Nutr 9:880488. 10.3389/fnut.2022.880488 35662952 10.3389/fnut.2022.880488 PMC9161358

[98] Yao M, Zhang H, Cai P, Gu X, Wang D, Ji Q (2016) Enhanced fitness of a *Bactrocera cucurbitae* genetic sexing strain based on the addition of gut-isolated probiotics (*Enterobacter* spec.) to the larval diet. Entomol Exp Appl 161:138–145. 10.1111/eea.12529

[99] Li G, Xiao Y, Leng J, Lou Q, Zhao T (2024) Beneficial efficacy and mode of action of probiotic *Bacillus subtilis* SWL–19 on the silkworm (*Bombyx mori* L). Symbiosis 93:17–27. 10.1007/s13199-024-00986-4

[100] Arora AK, Miller TA, Durvasula RV (2019) Transmission of *Pantoea agglomerans*—A paratransgenic control agent—within a *Homalodisca vitripennis* population. J Appl Entomol 143:1437–1442. 10.1111/jen.12720

[101] Lauzon CR, McCombs SD, Potter SE, Peabody NC (2009) Establishment and vertical passage of *Enterobacter (Pantoea) agglomerans* and *Klebsiella pneumoniae* through all life stages of the Mediterranean fruit fly (Diptera: Tephritidae). Ann Entomol Soc Am 102(1):85–95. 10.1603/008.102.0109

[102] Itoh H, Matsuura Y, Hosokawa T, Fukatsu T, Kikuchi Y (2017) Obligate gut symbiotic association in the sloe bug *Dolycoris baccarum* (Hemiptera: Pentatomidae). Appl Entomol Zool 52:51–59. 10.1007/s13355-016-0469-1

[103] Jucker C, Belluco S, Oddon SB, Ricci A, Bonizzi L, Lupi D, Savoldelli S, Biasato I, Caimi C, Mascaretti A, Gasco L (2022) Impact of some local organic by-products on *Acheta domesticus* growth and meal production. J Insects Food Feed 8:631–640. 10.3920/JIFF2021.0121

[104] Vaga M, Berggren Å, Jansson A (2021) Growth, survival and development of house crickets (*Acheta domesticus*) fed flowering plants. J Insects Food Feed 7:151–162. 10.3920/JIFF2020.0048

